# One Pot Synthesis of Micromolar BACE-1 Inhibitors Based on the Dihydropyrimidinone Scaffold and Their Thia and Imino Analogues

**DOI:** 10.3390/molecules25184152

**Published:** 2020-09-10

**Authors:** Jessica Bais, Fabio Benedetti, Federico Berti, Iole Cerminara, Sara Drioli, Maria Funicello, Giorgia Regini, Mattia Vidali, Fulvia Felluga

**Affiliations:** 1 Dipartimento di Scienze Chimiche e Farmaceutiche, Università di Trieste, via Licio Giorgieri 1, 34127 Trieste, Italy; JESSICA.BAIS@studenti.units.it (J.B.); benedett@units.it (F.B.); fberti@units.it (F.B.); sdrioli@units.it (S.D.); giorgiaregini@yahoo.it (G.R.); MATTIA.VIDALI@phd.units.it (M.V.); 2 Dipartimento di Scienze, Università della Basilicata, Viale dell’Ateneo Lucano 10, 85100 Potenza, Italy; iole.cerminara@gmail.com (I.C.); Maria.funicello@unibas.it (M.F.)

**Keywords:** Alzheimer’s disease, dihydropyrimidinones, Biginelli reaction, β-secretase, BACE-1 inhibitors

## Abstract

A library of dihydropyrimidinones was synthesized via a “one-pot” three component Biginelli reaction using different aldehydes in combination with β-dicarbonyl compounds and urea. Selected 2-thiooxo and 2-imino analogs were also obtained with the Biginelli reaction from thiourea and guanidine hydrochloride, respectively. The products were screened in vitro for their β-secretase inhibitory activity. The majority of the compounds resulted to be active, with IC_50_ in the range 100 nM–50 μM.

## 1. Introduction

**Scheme 1:**
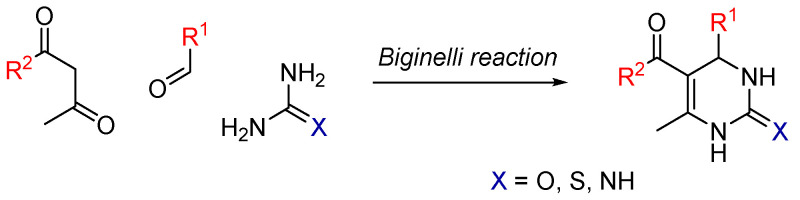
General scheme for the Biginelli reaction.

The 3,4-Dihydro-2-pyrimidinones (DHPMs), readily accessible in a single step via the Biginelli reaction [1,2,3] (Scheme 1, X = O), have recently attracted considerable attention for their multifaceted pharmacological properties [4,5]. Biologically active compounds based on this scaffold include HIV integrase inhibitors [6], human lactate hydrogenase inhibitors [7], calcium channel modulators [8], inhibitors of mitotic kinesin Eg5 [9], α_1A_ adrenoceptor antagonists [10], inhibitors of prostaglandin E_2_ synthase [11], and other compounds with anticancer, antibacterial, antiviral, anti-inflammatory, antihyperglycemic, and analgesic activity [4,12,13].

The varied activity of DHPMs, combined with their synthetic accessibility and the possibility to rapidly assemble diverse substituents on the heterocyclic scaffold in a single multicomponent reaction, make the Biginelli reaction an attractive approach for the discovery of new biological activities. Multicomponent reactions, in particular, show great promise in the discovery of small molecule protease inhibitors possessing better pharmacological properties than peptidomimetic inhibitors [14,15,16].

Recently, there has been increasing interest in the development of small, non-peptide inhibitors of the β-amyloid cleaving enzyme 1 (BACE-1, β-secretase) [17,18,19,20,21,22,23,24], following the suggestion that this enzyme may be involved in the pathogenesis of Alzheimer’s Disease (AD) [19,25]. BACE-1 is an aspartyl protease involved in the proteolytic degradation of the β-amyloid precursor protein (APP) to form amyloid-β peptide (Aβ), a small protein with a high tendency to form aggregates that are found in the AD brain [19]. Nanomolar inhibitors based on several heterocyclic scaffolds have been developed and a number have progressed to clinical trials [17,18,26]. Although some programs have been discontinued due to phase III failure, interest in BACE-1 inhibitors is still high, in particular for the development of modifying therapies for early stage AD [27,28].

A structural feature common to these scaffolds is the presence of functional groups capable of establishing hydrogen bond interactions with the enzyme’s catalytic aspartates [18,19]. DHPMs similarly possess neighboring hydrogen bond donor and acceptor groups; we thus decided to explore the potential of the Biginelli reaction for the synthesis of new β-secretase inhibitors. In this work we report on the synthesis and preliminary evaluation of a set of dihydropyrimidinones and their thia and aza analogues (Scheme 1, X=S, NH) as BACE-1 inhibitors.

The analysis of the BACE-1 active site in complex with crystallized inhibitors [18,19], allowed for a preliminary assessment of the potential of Biginelli products as inhibitors. The cyclic urea motif embedded in the DHMP scaffold and its thiourea and guanidine analogues are in principle capable to establish multiple hydrogen bond interactions with the dyad of catalytic aspartates (Asp 32, Asp 228), while it is possible to introduce diversity at R^1^ and R^2^ (Scheme 1), to allow interactions at least with the closer subsites S1 (wider) and S1′ (narrow), which are both hydrophobic. We therefore planned to synthesize a first set of potential inhibitors **1**–**17** reported in Table 1, bearing different aromatic or aliphatic residues at R^1^ (including also polar groups) and ester, amide, or free carboxylic groups at R^2^.

## 2. Results and Discussion

The synthetic work started with the preparation of 3,4-dihydropyrimidin-2(1*H*)-ones **1a**–**16a**, and the corresponding thiones **1b**–**3b**, **9b**, **10b**, **12b** and **16b** by the Biginelli reaction of aldehydes and β-dicarbonyl compounds with urea or thiourea, respectively (Table 1). A wide variety of experimental conditions have been described for this reaction, with different solvents and acid catalysts [2,3], including microwave irradiation [30], solid phase [31,32], and mechanochemical [33] methods. Following a solvent free protocol [34] we carried out the reactions at 100 °C for three hours in the presence of ammonium chloride, obtaining the target compounds with yields from 50% to 92%. The carboxylic acids **16a** and **16b** were obtained by NaOH hydrolysis of **1a** and **1b**, respectively. Most of these compounds were already known in the literature, except for **9a**, **10a**, and **10b**.

The inhibitory activity of the compounds thus obtained was determined in vitro on recombinant BACE-1 using a fluorogenic substrate analogue [35]. The results are reported in Table 1.

All the DHPMs and their thia derivatives inhibit BACE-1, with IC_50_s in the low μM range, except for **11a** (71.3 μM) and **16a** (34.0 μM). The most active compound among DHPMs is **2a**, with IC_50_ 0.32 μM. This inhibitor carries a phenyl group at R^1^, and a methyl group at R^2^. Compounds in which the phenyl group is replaced by other aromatic or aliphatic groups generally retain a significant IC_50_, although no improvement in activity is observed. No clear-cut electronic effect can be observed for aromatic systems with electron-withdrawing or donating groups (**3a**, **4a**, **5a**, and **8a**). Larger aromatic groups such as naphthyl (**9a**, **10a**) and benzothienyl (**11a**, **12a**) appear to be tolerated by the enzyme; in this case, orientation seems to be an important factor, as shown by the comparison between **9a** and **10a** and, much more so, between **11a** and **12a**. Inhibition appears to be more sensitive to substitution at R^2^: the methyl ester **2a** is more active than the corresponding ethyl ester **1a**, and amides **14a**, **15a,** while the presence of a free carboxylic group in **16a** lead to a major loss of activity. The latter may be related to the ionization of this molecule leading to mismatching interactions with the anionic catalytic site.

Thia derivatives, on average, are more active than DHPMs, and this is particularly evident in **16b**, where sulfur is able to restore the poor activity of **16a**. Better activities of sulfur containing compounds with respect to oxygen analogues are often observed in the interactions of small molecules with proteins. This has been explained with the reduced desolvation penalty that, in general, must be paid by sulfur compounds on entering a protein binding site [36].

Following on from the promising results observed with DHPMs and their 2-thia derivatives, we extended the study to a small selection of 2-imino-derivatives (Table 1: **1c**–**3c**, **9c**, **10c**, **16c**). The Biginelli condensation of guanidine has been much less investigated than the corresponding reactions with urea and thiourea. Kappe, in 2001 [37], reported the one-pot three-component reaction with guanidine hydrochloride, giving the cyclocondensation products in satisfactory yields; the method, however, was limited to α-benzoylethylacetate as the β-dicarbonyl partner. Reduced reaction times were recently reported under ultrasonic irradiation [38] while a general procedure for the Biginelli reaction using a phase transfer catalyst, compatible also with guanidine, has been described [39].

Ethyl and methyl acetoacetate reacted smoothly with aldehydes under microwave irradiation [29] (EtOH, MW 120 °C, 10 min, excess NaHCO_3_) to give the corresponding 2-iminodihydropyrimidines **1c**–**3c**, **9c** and **10c** with good yields. Finally, the acid **16c** was obtained by Pd/C hydrogenolysis of the corresponding benzyl ester **17c**. 3,4-Dihydro-pyrimidin-2(1*H*)-imine derivatives (or their 2-amino tautomers) can, in principle, establish better interactions with the enzyme’s active site than their 2-oxo or 2-thia analogues. The more basic guanidine system is protonated at the enzyme’s optimal pH and can thus interact with the catalytic aspartate dyad by both charge complementarity and an extended network of hydrogen bonds [40]. Accordingly, imine derivatives **1c**–**3c**, **9c**, **10c**, and **16c** are better inhibitors than their oxygen and thia analogs in nearly all cases. Only **9c** is less active than the corresponding thia-derivative **9b**, while **3b** and **3c** have comparable activities. Compound **10c** is the most active inhibitor overall showing a higher activity that the isomer **9c**; this confirms that the orientation of the large aromatic group with respect to the dihydropyrimidine group is important, as already observed for inhibitors **9a**, **10a 11a**, and **12a.** The improvement of the activity is very marked on going from **16a** to **16c**. In this case, however, the net charge of the urea, thiourea, and guanidine derivatives are different, as **16a** and **16b** are anions, while **16c** is, presumably, a neutral molecule at the enzyme’s optimal pH.

BACE-1 inhibitors must cross the blood-brain barrier in order to reach their target. Thus, permeability is an important factor to assess their pharmacological potential. BBB permeation is a complex process, depending on the interplay of several physicochemical parameters, among which lipophilicity, as defined by the octanol-water partition coefficient, is generally considered one of the most important [41]. Calculated LogP values (LogD for ionizable compounds) for inhibitors **1**–**16** (Table 1) show that, for several inhibitors, this value falls close to the 2.8 median value for marketed CNS drugs (1.7 for LogD) [41]. The more sophisticated BOILED-Egg model, taking into account also the polar surface area [42], assigns **3a**, **4a**, **9a**,**c** and **10a**,**c** as BBB+, thus predicting a good permeation for these compounds. These data suggest the possibility to develop the active compounds described in this paper towards more efficient inhibitors with favorable ADME properties.

## 3. Docking Analysis

**Figure 1:**
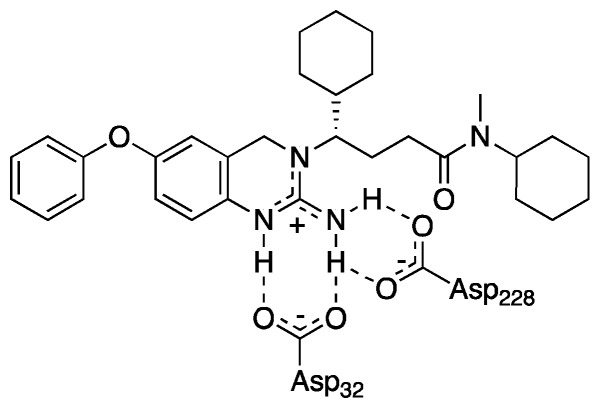
Baxter’s inhibitor and network of hydrogen bonds with the BACE-1 catalytic diad.

The IC_50_ values of Table 1 were obtained for the racemic products of the Biginelli reaction. To preliminary assess the affinity of enantiomerically pure inhibitors for the enzyme’s catalytic site, we have built computational models for a small set of guanidines by a molecular dynamics-based docking protocol. The set comprises both the (*R*) and (*S*) enantiomers of the most active compound **10c**, the (*R*) enantiomers of its isomer **9c**, and of compound **1c**. The models were built starting from the crystallographic structure of BACE-1 complexed with Baxter’s inhibitor [43]. This inhibitor (IC_50_ 11nM) has a guanidine fragment that, in its protonated form, establishes four hydrogen bonds with the enzyme’s catalytic aspartate diad (Figure 1).

The models were built by manually docking the protonated inhibitors, to the enzyme’s binding site and a starting geometry was obtained by superimposing the guanidinium group of each inhibitor onto that of the Baxter inhibitor in the reference complex. The starting models were then optimized by molecular dynamics and molecular mechanics with the OPLS3 forcefield. Binding energies ($\Delta E_{b}$, Equation (1)) were calculated as the difference between the energy of the enzyme-inhibitor complex (E_EI_) and the energies of the uncomplexed enzyme $(E_{E}^{0}$) and inhibitor $\left(E_{I}^{0}\right)$.

(1) $$\Delta E_{b}={\;E}_{\mathrm{EI}}-E_{E}^{0}-E_{I}^{0}$$

The resulting binding energies are reported in Table 2.

**Figure 2:**
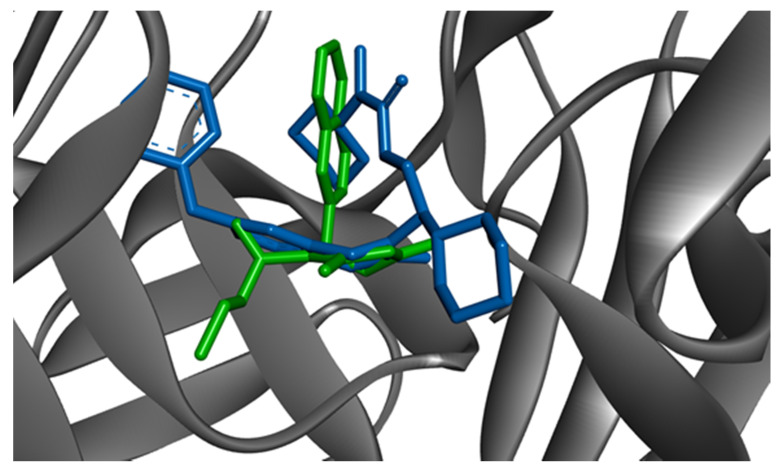
Overlay of the crystal structure of the complex of BACE-1 with the Baxter inhibitor (blue) and the best calculated pose of **(*R*)-10c** (green). The dihydropyrimidine rings of the two inhibitors are nearly perfectly superimposed; the naphthyl group of **(*R*)-10c** is superimposed to the terminal cyclohexyl group of Baxter inhibitor’s side chain and the ester group is partially superimposed to the fused benzene ring.

Inhibitor **(*R*)-10c** ranks first in binding energy within the series, and its pose inside the catalytic site resembles strictly that of the Baxter inhibitor (Figure 2), with the 2-naphthyl group well placed inside the S1 subsite of the catalytic site.

**Figure 3:**
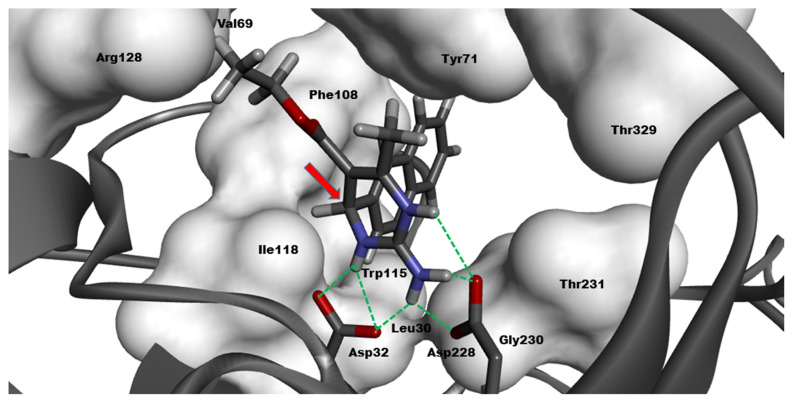
Best docking pose of **(*R*)**-**10c** and its interactions with BACE-1. Red arrow: the stereogenic center of the inhibitor.

The main interactions of **(*R*)-10c** are shown in Figure 3. The guanidinium system establishes a network of hydrogen bonds with the two aspartyl residues, resembling that of Baxter’s inhibitor. The 2-naphthyl group is at hydrophobic contact distance with most of the residues of the S1 subsite, namely Phe 108 and Tyr 71 on the upper flap, Leu 30, Trp 115, and Ile 118 at the bottom of the subsite. The ethyl ester points towards the surface of S3′, with some minor steric clashes that lead to a certain change in the local conformation of the flap. It is likely that these clashes are relieved in the smaller methyl esters and this may be the reason for the slight improvement of IC_50_ which is observed on going from the ethyl ester **1a** to the corresponding methyl ester **2a**.

The enantiomeric inhibitor **(*S*)-10c** is predicted to bind less favorably (Table 2). Actually, none of the poses found for this enantiomer leads to optimal interactions. With respect to the (*R*) enantiomer, the hydrogen atom and the naphthalene ring bonded to the stereogenic center (red arrow in Figure 3), exchange positions, and the large aromatic systems points towards the bottom residues of S1 (Asn 37, Leu 119, Ile 118), leading to severe steric clashes (Supplementary Figure S20). Any solution to this bumping leads to a loss in binding energy, either at the aspartyl residues level, or at the S1 subsite that cannot be filled by the naphthyl group in the same way as in the (*R*) enantiomer. The measured activity of racemic **10c** is thus likely due to the (*R*) enantiomer alone.

Having thus predicted that **(*R*)-10c** is a better binder than its **(*S*)**-enantiomer, the docking analyses on the remaining compounds were carried out for this configuration only. The 1-naphthyl derivative **9c**, is about one order of magnitude less active than **10c** (Table 1) and, accordingly, the calculated binding energy of **(*R*)-9c** is less favorable than that of **(*R*)-10c** by over 5 Kcal/mol (Table 2). In this case, the different orientation of the naphthyl system makes it impossible to preserve the optimal hydrogen bond network of the guanidinium system without clashes with Ile118 in the S1 subsite (Figure S21). Finally, in the complex with **(*R*)-1c**, the phenyl ring of the inhibitor occupies the same position in the catalytic site as the naphthyl ring of **(*R*)-10c**, but the hydrophobic contact with S1 is reduced due to its smaller size. This results in a slightly less favorable binding energy (Table 2), in agreement with the observed lower activity of **1c**.

In conclusion, we show here that 3,4-dihydro-(1*H*)-pyrimidin-2-ones (DHPMs) and their 2-thia and 2-imino derivatives, easily obtained in one pot by the Biginelli reaction, show promising inhibitory activity against β-secretase. Notwithstanding the simple structure, that only allows interactions with the enzyme’s S1 and S3′ subsites (Figure 3), most of the compounds synthesized in this work inhibit BACE-1 at the μM and sub-μM level. Docking studies reveal that (*R*) enantiomers fit better in the enzyme’s binding site than (*S*) enantiomers and thus should be better inhibitors. This would imply that the activities observed for the racemic mixtures (Table 1) are due, mostly or exclusively, to one enantiomer. However, as was pointed out by a referee, the contributions of enantiomers may vary to some extent. This may explain deviations from a general trend, as observed, for example, in compounds **2** and **10**, where the sulfur derivatives **2b** and **10b** are less active than the corresponding oxygen compounds **2a** and **10a**. The development of this approach toward more efficient inhibitors is currently underway in our laboratory and includes the synthesis of enantiomerically pure inhibitors and the introduction of substituents on the scaffold’s accessible positions, in order to target the other enzyme’s subsites.

## 4. Materials and Methods

### 4.1. General Experimental Information

NMR spectra were recorded on a Varian 500 spectrometer (Palo Alto, CA, USA) in DMSO-d_6_ or in MeOD, at 500 MHz (^1^H) and 125.68 MHz (^13^C); chemical shifts are in ppm (δ) with DMSO (δ = 2.50 for ^1^H-NMR and 39.50 for ^13^C-NMR) or with MeOD (δ = 3.34 for ^1^H-NMR and 47.60 for ^13^C-NMR) as the reference. Coupling constants J are given in Hertz. ^1^H and ^13^C-NMR resonances were assigned using a combination of DEPT, COSY, and HSQC spectra. Infrared (IR) spectra were recorded as thin film or Nujol mull on NaCl plates on a Nicolet Avatar FT-IR spectrometer (Thermo Fisher Scientific, Waltham, MA, USA). Melting points are uncorrected. Electrospray (ESI) mass spectra were obtained on a Bruker Daltonics Esquire 4000 spectrometer (Billerica, MA, USA). HRMS were obtained on a Bruker micrOTOF-Q. Fluorimetric assays were run on a Perkin Elmer LS50B spectrofluorimeter (Waltham, MA, USA). Yields refer to spectroscopically (^1^H-NMR) homogeneous materials. Commercial reagents and solvents were purchased from Sigma-Aldrich.

### 4.2. Synthesis and Characterization

#### 4.2.1. General Method for the Synthesis of 3,4-Diihydropyrimidin-2(1*H*)-Ones **1a**–**16a** and 3,4-Dihydropyrimidin-2(1*H*)-Thiones **1b**–**3b**; **9b**,**10b**; **13b**

Following a literature procedure [34], a mixture of the appropriate aldehyde (1 mmol), β-ketoester, or β-ketoamide (1 mmol), urea or thiourea (1.5 mmol) and NH_4_Cl (0.4 mmol) was heated with stirring at 100 °C for 3 h. After cooling, cold water was added (25 mL) and the resulting solid was recrystallized from ethyl acetate/n-hexane (1:3).

*5-Ethoxycarbonyl-6-methyl-4-phenyl-3,4-dihydropyrimidin-2(1H)-one***1a** [44]. From benzaldehyde, ethylacetoacetate, urea; 0.213 g, 82%, white solid, m.p. 205–206 °C; IR: 3420, 3220, 1724, 1700, 1646 cm^−1^; ^1^H-NMR (500 MHz, DMSO-d_6_): δ 1.09 (t, 3H, J = 10.0 Hz, CH_3_CH_2_O), 2.24 (s, 3H, C(6)CH_3_), 3.91 (q, 2H, CH_3_CH_2_O), 5.14 (d, 1H, J = 2.5 Hz, H-4), 7.20–7.36 (m, 5H, PhH), 7.74 (s, 1H, H-3), 9.20 (s, 1H, H-1) ppm; ^13^C-NMR (125.68 MHz, DMSO-d_6_): δ 14.0, 17.8, 54.0, 59.3, 99.4, 126.4, 127.5, 128.6, 145.1, 148.6, 152.4, 165.6 ppm; ESI-MS, m/z: 261.5 [M+H]^+^, 283.4 [M+Na]^+^; Anal. Calcd. for C_14_H_16_N_2_O_3_ C, 64.60; H, 6.20; N, 10.76; Found: C, 64.71; H, 6.18; N, 10.70.

*5-Methoxycarbonyl-6-methyl-4-phenyl-3,4-dihydropyrimidin-2(1H)-one***2a** [33]. From benzaldehyde, methylacetoacetate, urea; 0.204 g, 83%, white solid, m.p. 207–209 °C; IR: 3420, 3220, 1693, 1642 cm^−1^; ^1^H-NMR (500 MHz, DMSO-d_6_): δ 2.25 (s, 3H, C(6)CH_3_), 3.53 (s, 3H, CH_3_O), 5.14 (d, J = 1.0 Hz, 1H, H-4), 7.23–7.34 (m, 5H, Ph), 7.74 (s, 1H, H-3), 9.20 (s, 1H, H-1); ^13^C-NMR (125.68 MHz, DMSO-d_6_): δ 17.9, 50.7, 53.7, 99.0, 126.2, 127.3, 128.3, 144.7, 148.7, 152.2, 165.8 ppm; ESI/MS, m/z: 269.4 [M+Na]^+^; Anal. Calcd. for C_13_H_14_N_2_O_3_ C, 63.40; H, 5.73; N, 11.38; Found: C, 63.51; H, 5.68; N, 11.45.

*5-Ethoxycarbonyl-4-(4-fluorophenyl)-6-methyl-3,4-dihydropyrimidine-2(1H)-one***3a** [44]. From 4-fluorobenzaldehyde, ethylacetoacetate, urea; 0.195 g, 70%, yellow solid, m.p. 199–200 °C; IR: 3285, 2374, 1700, 1088 cm^−1^; ^1^H-NMR (500 MHz, DMSO-d_6_): δ 1.09 (t, 3H, J = 7.2 Hz, CH_3_CH_2_O), 2.25 (s, 3H, C(6)CH_3_), 3.98 (q, 2H, J = 7.2 Hz, CH_3_CH_2_O), 5.15 (d, 1H, J = 4.5 Hz, H-4), 7.11-7.18 (m, 2H, PhH), 7.24–7.29 (m, 2H, PhH), 7.75 (s, 1H, H-3), 9.22 (s, 1H, H-1) ppm. ^13^C-NMR (125.68 MHz, DMSO-d_6_): δ 14.1, 17.8, 53.3, 59.2, 99.1, 115.1 (d, ^2^J_CF_ = 21.0 Hz), 128.2 (d, ^3^J_CF_ = 8.0 Hz), 141.1 (d, ^4^J_CF_ = 3.0 Hz), 150.3 (d, ^1^J_CF_ = 345.0 Hz), 160.1, 165.2 ppm. ESI/MS, m/z: 279.1 [M+H]^+^; Anal. Calcd. for C_14_H_15_FN_2_O_3_ C, 60.43; H, 5.43; N, 10.07; Found: C, 60.31; H, 5.28; N, 10.27.

*5-Ethoxycarbonyl-4-((4-trifluoromethyl)phenyl)-6-methyl-3,4-dihydropyrimidine-2(1H)-one***4a** [45]. From 4-trifluoromethylbenzaldehyde, ethylacetoacetate, urea; 0.266 g, 81%, m.p. 177–179 °C; IR: 3285, 2374, 1705, 1086 cm^−1^; ^1^H-NMR (500 MHz, DMSO-d_6_): δ 1.08 (t, 3H, J = 7.2 Hz, CH_3_CH_2_O), 2.26 (s, 3H, C(6)CH_3_), 3.98 (q, 2H, J = 7.2 Hz, CH_3_CH_2_O), 5.23 (d, 1H, J = 1.0 Hz, H-4), 7.45 (2H, o-CF_3_-ArH), 7.70 (2H, m-CF_3_-ArH), 7.74 (s, 1H, H-3), 9.23 (s, 1H, H-1) ppm; ^13^C-NMR (125.68 MHz, DMSO-d_6_): δ 14.05, 17.8, 53.7, 59.3, 98.5, 125.45 (q, ^3^J_CF_ = 5.0 Hz), 127.1, 127.9 (d, ^2^J_CF_ = 19.0 Hz), 126.4 (q, ^1^J_CF_ = 193.0 Hz), 149.1, 149.3, 151.89, 165.1 ppm. ESI/MS, m/z: 329.1 [M+H]^+^; Anal. Calcd. for C_15_H_15_F_3_N_2_O_3_ C, 54.88; H, 4.61; N, 8.53; Found C, 55.02; H, 4.49; N, 8.58.

*5-Ethoxycarbonyl 4-(4-hydroxy-3-methoxyphenyl)-6-methyl-3,4-dihydropyrimidine-2(1H)-one***5a** [44]. From 4-hydroxy-3-methoxybenzaldehyde, ethylacetoacetate, urea; 0.239 g, 78%, yellow solid, m.p. 228–230 °C; IR: 3248, 3114, 1701, 1647, 1518 cm^−1^; ^1^H-NMR (500 MHz, DMSO-d_6_): δ 1.11 (t, 3H, J = 10.0 Hz, CH_3_CH_2_O), 2.23 (s, 3H, C(6)CH_3_), 3.72 (s, 3H, CH_3_O), 3.99 (q, 2H, J = 9.0 Hz, CH_3_CH_2_O), 5.05 (d, 1H, J = 4.0 Hz, H-4), 6.55-6.83 (m, 3H, ArH), 7.62 (bs, 1H, H-3), 8.89 (s, 1H, OH), 9.11 (s, 1H, H-1) ppm; ^13^C-NMR (125.68 MHz, DMSO-d_6_): δ 14.2, 17.7, 53.6, 55.6, 59.1, 99.5, 135.9, 145.8, 147.2, 147.9, 152.2, 165.5 ppm; ESI-MS, m/z: 307.0 [M+H]^+^; Anal. Calcd. for C_15_H_18_N_2_O_5_ C, 58.82; H, 5.92; N, 9.15; Found C, 58.64; H, 5.95; N, 8.96.

*5-Ethoxycarbonyl-4-(thiophen-3-yl)-6-methyl-3,4-dihydropyrimidin-2(1H)-one***6a** [46]. From 3-thiophenecarboxaldehyde, ethylacetoacetate, urea; 0.154 g, 58%, yellow solid. m.p. 230–232 °C; ^1^H-NMR (500 MHz): δ 1.14 (t, 3H, J = 7.2 Hz, CH_3_CH_2_O), 2.20 (s, 3H, C(6)CH_3_), 4.03 (q, 2H, J = 7.2 Hz, CH_3_CH_2_O), 5.19 (d, 1H, J = 3.2 Hz, C(4)H), 6.97 (d, 1H, J = 4.8 Hz, H-4′), 7.14 (s, 1H, H-2′), 7.45 (d, 1H, J = 4.8 Hz, H-5′), 7.75 (bs, 1H, H-3); 9.19 (s, 1H, H-1) ppm; ^13^C-NMR (125.68 MHz): δ 14.5, 18.1, 49.7, 59.6, 99.8, 121.1, 126.5, 127.0, 146.1, 148.8, 153.0, 165.0 ppm; HRMS-ESI, m/z (**S7**): 355.0520 [M+K]^+^; Calcd. for [C_16_H_16_KN_2_O_3_S]^+^: 355.0513; ESI/MS, m/z: 267.1 [M+H]^+^; Anal. Calcd. for C_12_H_14_N_2_O_3_S C, 54.12; H, 5.30; N, 10.52; Found C, 54.00; H, 5.45; N, 10.43.

*5-Ethoxycarbonyl-4-(thiophen-2-yl)-6-methyl-3,4-dihydropyrimidin-2(1H)-one***7a** [46]. From 2-thiophenecarboxaldehyde, ethylacetoacetate, urea; 0.162 g, 61%, yellow solid, m.p. 220–222 °C; ^1^H-NMR (500 MHz, DMSO-d_6_): δ 1.15 (t, 3H, J = 7.2 Hz, CH_3_CH_2_O), 2.20 (s, 3H, C(6)CH_3_), 4.05 (q, 2H, J = 7.2 Hz, CH_3_CH_2_O), 5.40 (d, 1H, J = 3.2 Hz, H-4), 6.87-6.89 (m, 2H, H-3′ and H-4′), 7.35 (m, 1H, H-5′), 7.90 (s, 1H, H-3), 9.31 (s, 1H, H-1) ppm; ^13^C-NMR (125.68 MHz, DMSO-d_6_): δ 14.6, 18.1, 49.8, 59.8, 79.6, 100.2, 124.0, 125.1, 127.15, 149.1, 152.7, 165.5. ppm; ESI/MS, m/z: 267.1 [M+H]^+^; 289.1 [M+Na]^+^; Anal. Calcd. for C_12_H_14_N_2_O_3_S: C, 54.12; H, 5.30; N, 10.52; Found C, 54.10; H, 5.41; N, 10.40.

*5-Ethoxycarbonyl-4-(5-nitrothiophen-2-yl)-6-methyl-3,4-dihydropyrimidin-2(1H)-one***8a.** From 5-nitro-2-thiophenecarboxaldehyde, ethylacetoacetate, urea; 0.156 g, 50%, yellow solid, m.p. 221–222 °C; ^1^H-NMR (500 MHz) (Figure S2): δ 1.17 (t, 3H, J = 7.2 Hz, CH_3_CH_2_O), 2.23 (s, 3H, C(6)CH_3_), 4.09 (q, 2H, J = 7.2 Hz, CH_3_CH_2_O), 5.39 (d, 1H, J = 2.2 Hz, H-4), 7.01 (d, 1H, J = 4.3 Hz, H-3′), 7.98 (d, 1H, J = 4.3 Hz, H-4′), 8.13 (bs, 1H, H-3), 9.53 (s, 1H, H-1) ppm; ^13^C-NMR (125.68 MHz) (Figure S3): δ 14.6, 18.2, 50.2, 60.2, 98.6, 124.5, 130.5, 149.5, 150.7, 152.3, 158.2, 165.2 ppm; HRMS-ESI, m/z (Figure S4): 312.0647 [M+H]^+^; Calcd. For [C_12_H_14_N_3_O_5_S]^+^: 312.0649; Anal. Calcd. for C_12_H_13_N_3_O_5_S C, 46.30; H, 4.21; N, 13.50; Found C, 46.43; H, 4.20; N, 13.43.

*5-Ethoxycarbonyl-6-methyl-4-(naphthalen-1-yl)-3,4-dihydropyrimidin-2(1H)-one***9a** [47,48]. From 1-naphtaldehyde, ethylacetoacetate, urea; 0.276 g, 89%, white solid, m.p. 251–252 °C; IR: 3239, 3110, 2987, 2931, 1707, 1649, 1467, 1315, 1223, 1086, 793, 779 cm^−1^; ^1^H-NMR (500 MHz, DMSO-d_6_): δ 0.81 (t, 3H, J = 7.0 Hz, CH_3_CH_2_O), 2.35 (s, 3H, C(6)CH_3_), 3.80 (m, 2H, CH_3_CH_2_O), 6.06 (d, 1H, J = 3.1, H-4), 7.41 (d, 1H, ArH), 7.74 (bs, 1H, H-3), 7.46-7.62 (m, 3H, ArH), 7.94 (d, 1H, ArH), 8.02 (d, 1H, ArH), 8.30 (d, 1H, ArH), 9.25 (s, 1H, H-1) ppm. ^13^C-NMR (125.68 MHz, DMSO-d_6_): δ 13.8, 17.8, 49.8, 59.0, 99.1, 123.6, 124.2, 125.6, 125.7, 126.0, 127.9, 128.5, 133.5, 140.4, 148.7, 151.6, 157.3, 165.3 ppm; ESI/MS, m/z 311.4 [M+H]^+^ 333.4 [M+Na]^+^; Anal. Calcd. for C_18_H_18_N_2_O_3_ C, 69.66; H, 5.85; N, 9.03; Found C, 69,81; H, 5.66; N, 9.13.

*5-Ethoxycarbonyl-6-methyl-4-(naphthalen-2-yl)-3,4-dihydropyrimidin-2(1H)-one***10a** [47]. From 2-naphtaldehyde, ethylacetoacetate, urea; 0.270 g, 87%, yellow solid, m.p. 211–213 °C; IR: 3247, 3123, 2975, 1719, 1658, 1459, 1292, 1226, 1088 cm^−1^; ^1^H-NMR (500 MHz, DMSO-d_6_): δ 1.08 (t, 3H, J = 7.0 Hz, CH_3_CH_2_O), 2.29 (s, 3H, C(6)CH_3_), 3.97 (q, 2H, J = 7.0 Hz, CH_3_CH_2_O), 5.32 (d, 1H, J = 3.0, H-4), 7.42–7.53 (m, 3H, ArH), 7.67 (s, 1H, ArH), 7.82 (bs, 1H, H-3), 7.85-7.91 (m, 3H, ArH), 9.24 (s, 1H, H-1) ppm; ^13^C-NMR (125.68 MHz, DMSO-d_6_): δ 14.1, 17.9, 54.3, 59.2, 99.0, 124.6, 124.9, 125.9, 126.3, 127.5, 127.8, 128.3, 132.3, 132.7, 142.15, 148.5, 152.01, 165.3 ppm; ESI/MS, m/z: 311 [M+H]^+^; 333.5 [M+Na]^+^; Anal. Calcd. for C_18_H_18_N_2_O_3_ C, 69.66; H, 5.85; N, 9.03; Found C, 69.60; H, 5.96; N, 9.08.

*5-Ethoxycarbonyl-4-(benzo[b]thiophen-3-yl)-6-methyl-3,4-dihydropyrimidin-2(1H)-one***11a**. From benzo[b]thiophene-3-carboxaldehyde, ethylacetoacetate, urea; 0.231 g, 73%, yellow solid, m.p. 195–197 °C; ^1^H-NMR (500 MHz, DMSO-d_6_) (Figure S5): 0.98 (t, J = 7.2 Hz, 3H, CH_3_CH_2_O), 2.30 (s, 3H, C(6)CH_3_), 3.92 (m, 2H, CH_3_CH_2_O), 5.58 (s, 1H, C(4)H), 7.33-7.41 (m, 2H, ArH), 7.42 (s, 1H, thiophenyl CH=C), 7.82 (dd, J = 2.0 and 3.4 Hz, 1H, H-3), 7.96 (m, 2H, ArH), 9.29 (d, J = 2.0 Hz, 1H, H-1); ^13^C-NMR (125.68 MHz, DMSO-d_6_) (Figure S6): δ 14.48, 18.26, 48.81, 59.60, 98.75, 122.63, 123.44, 124.46, 124.76, 137.33, 139.30, 140.71, 149.28, 152.44, 165.65 ppm; HRMS-ESI, m/z (Figure S7): Found: 355.0520 [M+K]^+^; Calcd for [C_16_H_16_N_2_O_3_SK]^+^: 355.0513; Anal. Calcd. for C_16_H_16_N_2_O_3_S C, 60.74; H, 5.10; N, 8.85; Found C, 60.70; H, 5.26; N, 8.91.

*5-Ethoxycarbonyl-4-(benzo[b]thiophen-2-yl)-6-methyl-3,4-dihydropyrimidin-2(1H)-one***12a**. From benzo[b]thiophene-2-carboxaldehyde, ethylacetoacetate, urea; 0.269 g, 85%, white solid, m.p. 260–262 °C (dec.); IR: 3188, 3091, 2916, 1704, 1645, 1285, 1221, 1089 cm^−1^; ^1^H-NMR (500 MHz, DMSO-d_6_) (Figure S8): δ 1.19 (t, 2H, J = 7.1 Hz, CH_3_CH_2_O), 2.25 (s, 3H, C(6)CH_3_), 4.09 (q, 3H, J = 7.1 Hz, CH_3_CH_2_O), 5.50 (d, 1H, J = 3.5 Hz, H-4), 7.19 (s, 1H, thiophenyl CH=C), 7.28–7.36 (m, 2H, ArH), 7.79 (d, 1H, J = 7.2 Hz, ArH), 7.88 (d, 1H, J = 7.2 Hz, ArH), 8.01 (bs, 1H, H-3), 9.39 (s, 1H, H-1); ^13^C-NMR (125.68 MHz, DMSO-d_6_) (Figure S9): δ 14.2, 17.7, 50.0, 59.5, 99.0, 119.9, 122.5, 123.5, 124.2, 124.4, 138.6, 139.0, 149.2, 149.25, 152.2, 159.6, 164.0 ppm; HRMS, m/z (Figure S10): Found: 339.0784 [M+Na]^+^; Calcd for [C_16_H_16_N_2_O_3_SNa]^+^: 339.0774; Found: 355.0511 [M+K]^+^; Calcd for: [C_16_H_16_N_2_O_3_SK]^+^: 355.0513; Anal. Calcd. for C_16_H_16_N_2_O_3_S C, 60.74; H, 5.10; N, 8.85; Found C, 60.60; H, 5.16; N, 9.00.

*4-n-Butyl-5-ethoxycarbonyl-6-methyl-3,4-dihydropyrimidin-2(1H)-one***13a [49]**. From pentanal, ethylacetoacetate, urea; 0.120 g, 50%, white solid, m.p. 178–183 °C; IR: 3255, 3123, 2938, 1720, 1655 cm^−1^; ^1^H-NMR (500 MHz, DMSO-d_6_): δ 0.83 (t, 3H, J = 8.5 Hz, CH_3_(CH_2_)_3_, 1.16 (t, 3H, J = 9.0 Hz, CH_3_CH_2_O), 1.22 (m, 4H, CH_3_(CH_2_)_2_CH_2_), 1.36 (m, 2H, CH_3_(CH_2_)_2_CH_2_), 2.14 (s, 3H, C(6)CH_3_), 4.01 (m, 3H, CH_3_CH_2_O and H-4), 7.29 (bs, 1H, H-3), 8.90 (s, 1H, H-1) ppm; ^13^C-NMR (125.68 MHz, DMSO-d_6_): δ 14.0, 14.2, 17.7, 21.9, 25.9, 36.4, 50.0, 59.0, 99.4, 148.3, 152.8, 165.4 ppm; ESI-MS, m/z: 241.4 [M+H]^+^, 263.2 [M+Na]^+^, 279.3 [M+K]^+^; Anal. Calcd. for C_12_H_20_N_2_O_3_ C, 59.98; H, 8.39; N, 11.66; Found C, 60.12; H, 8.20; N, 11.70.

*5-Aminocarbonyl-6-methyl-2-oxo-4-phenyl-3,4-dihydropyrimidin-2(1H)-one***14a** [50]. From benzaldehyde, acetoacetamide, urea; 0.109 g, 47%, white solid, m.p. 220–222 °C; ^1^H-NMR (500 MHz, DMSO-d_6_): δ 2.06 (s, 3H, C(6)CH_3_), 5.30 (d, 1H, J = 3.0 Hz, H-4), 6.90 (bs, 2H, NH_2_), 7.21–7.35 (m, 6H, Ph), 7.49 (bs, 1H, H-3), 8.55 (s, 1H, H-1) ppm; ^13^C-NMR (125.68 MHz, DMSO-d_6_): δ 17.5, 55.1, 126.8, 127.6, 128.75, 139.2, 144.9, 153.1, 168.6 ppm; ESI-MS, m/z: 232.1 [M+H]^+^; Anal. Calcd. for C_12_H_13_N_3_O_2_ C, 62.33; H, 5.67; N, 18.17; Found C, 62.40; H, 5.72; N, 18.10.

*6-Methyl-4-phenyl-5-(piperidine-1-carbonyl)-3,4-dihydropyrimidin-2(1H)-one***15a** [50,51]. From benzaldehyde, 1-(piperidin-1-yl)-1,3-butanedione, prepared as in ref. [52], urea; 0.180 g, 60%, white solid, m.p. 267–269 °C; ^1^H-NMR (400 MHz, CDCl_3_): δ 1.10 (s, 2H, CH_2_), 1.33 (s, 2H, CH_2_), 2.95 (s, 4H, CH_2_NCH_2_), 5.17 (s, 1H, H-4), 7.19–7.35 (m, 5H, PhH), 8.44 (br, 1H, H-3) ppm; ^13^C-NMR (100 MHz, CDCl_3_): δ 15.7, 23.8, 25.2, 57.2, 100.8, 104.0, 126.3, 127.6, 128.0, 128.7, 144.2, 152.7, 166.7 ppm. ESI-MS, m/z: 322.2 [M+Na]^+^; Anal. Calcd. for C_17_H_21_N_3_O_2_ C, 68.20; H, 7.07; N, 14.04; Found C, 68.40; H, 6.97; N, 13.96.

*5-Carboxy-6-methyl-4-phenyl-3,4-dihydropyrimidin-2(1H)-one***16a** [51,53]. By NaOH hydrolysis of the ethylester (**1a**), following a reported procedure [10,12]. 0.105 g, 45%, white solid, m.p. 210–211 °C; IR: 3433, 3227, 1706, 1645 cm^−1^. ^1^H-NMR (500 MHz, DMSO-d_6_): δ 2.22 (s, 3H, C(6)CH_3_), 5.10 (d, J = 4.0 Hz, 1H, H-4), 7.18-7.34 (m, 5H, PhH), 7.64 (bs, 1H, H-3), 9.05 (s, 1H, H-1), 11.83 (s, 1H, COOH); ^13^C-NMR (125.68 MHz, DMSO-d_6_): δ 18.2, 54.4, 100.25, 126.7, 127.6, 128.8, 145.3, 148.2, 152.8, 167.6 ppm. ESI/MS, m/z: 233.1 [M+Na]^+^; Anal. Calcd. for C_12_H_12_N_2_O_3_ C, 62.06; H, 5.21; N, 12.06; Found C, 62.24; H, 5.12; N, 12.10.

*5-Ethoxycarbonyl-6-methyl-4-phenyl-3,4-dihydropyrimidin-2(1H)-thione***1b** [48]. From benzaldehyde, ethylacetoacetate, thiourea; 0.235 g, 85%, white solid, m.p. 203–204 °C; IR: 3356, 3251, 1668, 1573, 1462, 1283, 1195, 1117 cm^−1^; ^1^H-NMR (500 MHz, DMSO-d_6_): δ 1.10 (t, 3H, J = 7.0 Hz, CH_3_CH_2_O), 2.29 (s, 3H, C(6)CH_3_); 4.01 (q, 2H, J = 7.0 Hz, CH_3_CH_2_O); 5.17 (d, 1H, J = 3.5 Hz, H-4); 7.21-7.36 (m, 5H, PhH), 9.64 (bs, 1H, H-3); 10.32 (s, 1H, H-1) ppm; ^13^C-NMR (125.68 MHz, DMSO-d_6_): δ 14.5, 18.2, 54.5, 60.0, 101.1, 128.8, 128.1, 129.0, 143.9, 145.5, 165.6, 174.7 ppm; ESI-MS, m/z: 277.4 [M+H]^+^; 299.4 [M+Na]^+^; Anal. Calcd. for C_14_H_16_N_2_O_2_S C, 60.85; H, 5.84; N, 10.14; Found C, 60.80; H, 5.97; N, 10.10.

*5-Methoxycarbonyl-6-methyl-4-phenyl-3,4-dihydropyrimidin-2(1H)-thione***2b** [48]. From benzaldehyde, methylacetoacetate, thiourea; 0.228 g, 87 %, light yellow solid, m.p. 205–207 °C; IR: 1711, 1572, 1459, 1318, 1280, 1180, 1113 cm^−1^; ^1^H-NMR (500 MHz, DMSO-d_6_): δ 2.29 (s, 3H, C(6)CH_3_), 3.56 (s, 3H, CH_3_O), 5.18 (d, 1H, J = 3.5 Hz, H-4), 7.21–7.36 (m, 5H, PhH), 9.66 (bs, 1H, H-3), 10.35 (s, 1H, H-1) ppm; ^13^C-NMR (125.68 MHz, DMSO-d_6_): δ 17.2, 51.1, 53.9, 100.4, 126.3, 127.7, 128.6, 143.3, 145.3, 165.6, 174.25 ppm; ESI-MS, m/z: 263.4 [M+H]^+^; 285.4 [M+Na]^+^. Anal. Calcd. for C_13_H_14_N_2_O_2_S C, 59.52; H, 5.38; N, 10.68; Found C, 59.40; H, 5.47; N, 10.81.

*5-Ethoxycarbonyl-4-(4-fluorophenyl)-6-methyl-3,4-dihydropyrimidine-2(1H)-thione***3b** [46]. From 4-fluorobenzaldehyde, ethylacetoacetate, thiourea; 0.200 g, 68%, light yellow solid, m.p. 205–206 °C; IR: 3391, 3248, 1711, 1586 cm^−1^; ^1^H-NMR (400 MHz, DMSO-d_6_): δ 1.09 (t, 3H, J = 7.0 Hz, CH_3_CH_2_O), 2.29 (s, 3H, C(6)CH_3_), 4.00 (m, 2H, CH_3_CH_2_O), 5.17 (d, 1H, J = 4.0 Hz, H-4), 7.11–7.29 (m, 5H, ArH), 9.65 (bs, 1H, H-3), 10.36 (s, 1H, H-1) ppm; ^13^C-NMR (100 MHz, DMSO-d_6_): δ 14.0, 17.2, 53.4, 59.6, 100.6, 115.3 (d, ^2^J = 17.0 Hz), 128.4 (d, ^3^J = 7.0 Hz), 139.8 (d, ^4^J = 2.0 Hz), 161.5 (d, ^1^J = 194.0 Hz), 165.0, 174.2 ppm; ESI-MS, m/z: 295 [M+H]^+^; Anal. Calcd. for C_14_H_15_FN_2_O_2_S C, 57.13; H, 5.14; N, 9.52; Found C, 57.33; H, 5.087; N, 9.41.

*5-Ethoxycarbonyl-6-methyl-4-(naphth-1-yl)-3,4-dihydropyrimidin-2(1H)-thione***9b** [48]. From 1-naphtaldehyde, ethylacetoacetate, thiourea; 0.284 g, 87%, yellow powder, m.p. 223–225 °C; IR: 3427, 3208, 2967, 1695, 1562, 1454, 1334, 1210, 1143, 1021 cm^−1^. ^1^H-NMR (500 MHz, DMSO-d_6_): δ 0.83 (t, 3H, J = 7.0 Hz, CH_3_CH_2_O), 2.39 (s, 3H, C(6)CH_3_), 3.83 (m, 2H, CH_3_CH_2_O), 5.08 (d, 1H, J = 3.5 Hz, H-4), 7.38 (d, J = 7.0 Hz, 1H, ArH), 7.55 (m, 3H, ArH), 7.88 (d, J = 8.0 Hz, 1H, ArH), 7.95 (d, J = 8.0 Hz, 1H, ArH), 8.37 (d, J = 8.0 Hz, 1H, ArH), 9.64 (bs, 1H, H-3), 10.37 (s, 1H, H-1) ppm; ^13^C-NMR (125.68 MHz, DMSO-d_6_): δ 13.7, 17.1, 14.1, 49.7, 59.4, 100.8, 130.0, 133.4, 139.2, 145.3, 165.0, 173.7 ppm; ESI-MS, m/z: 327.3 [M+H]^+^, 349.2 [M+Na]^+^, 365.1 [M+K]^+^; Anal. Calcd. for C_18_H_18_N_2_O_2_S C, 66.23; H, 5.56; N, 8.58; Found C, 66,43; H, 5.56; N, 8.62.

*5-Ethoxycarbonyl-6-methyl-4-(naphth-2-yl)-3,4-dihydropyrimidin-2(1H)-thione***10b** [49]. From 2-naphtaldehyde, ethylacetoacetate, thiourea; 0.287 g, 88%, yellow solid, m.p. 181–183 °C; IR: 3435, 3209, 3097, 2961, 1693, 1559, 1461, 1335, 1214, 1146, 1023 cm^−1^; ^1^H-NMR (500 MHz, DMSO-d_6_): δ 1.11 (t, 3H, J = 7.0 Hz, CH_3_CH_2_O), 2.33 (s, 3H, C(6)CH_3_), 4.00 (m, 2H, CH_3_CH_2_O), 5.34 (d, J = 3.5 Hz, H-4), 7.45 (m, 1H, ArH), 7.54 (m, 2H, ArH), 7.71 (s, 1H, ArH), 7.93 (m, 1H, ArH), 7.96 (m, 3H, ArH), 9.73 (bs, 1H, H-3), 10.37 (s, 1H, H-1) ppm; ^13^C-NMR (125.68 MHz, DMSO-d_6_): δ 14.5, 17.7, 54.8, 60.0, 100.9, 125.2, 125.4, 126.9, 126.6, 128.0, 128.3, 129.0, 132.9, 133.1, 141.2, 145.7, 165.6, 174.6 ppm; ESI-MS, m/z: 327 [M+H]^+^ 349 [M+Na]^+^; Anal. Calcd. for C_18_H_18_N_2_O_2_S C, 66.23; H, 5.56; N, 8.58; Found C, 66.37; H, 5.50; N, 8.51.

*4-(Benzo[b]thiophen-2-yl)-5-ethoxycarbonyl-6-methyl-3,4-dihydropyrimidin-2(1H)-thione***12b**. From benzo[b]thiophene-2-carboxaldehyde, ethylacetoacetate, thiourea; 0.243 g, 73%, pale orange solid, m.p. 213 °C (dec); IR: 3272, 3167, 2978, 1705, 1558, 1310, 1182, 1100 cm^−1^; ^1^H-NMR (500 MHz, DMSO-d_6_) (Figure S11): δ 1.19 (t, 2H, J = 7.1 Hz, CH_3_CH_2_O), 2.30 (s, 3H, C(6)CH_3_), 4.11 (q, 3H, J = 7.1 Hz, CH_3_CH_2_O), 5.52 (d, 1H, J = 3.5 Hz, H-4), 7.19 (s, 1H, thiophenyl CH = C), 7.29–7.38 (m, 2H, ArH), 7.81 (d, J = 8.2 Hz, 1H, ArH), 7.90 (d, J = 8.2 Hz, 1H, ArH), 9.87 (s, 1H, H-3), 10.56 (s, 1H, H-1) ppm; ^13^C NMR (125.68 MHz, DMSO-d_6_) (Figure S12): δ 14.1, 17.1, 50.0, 59.8, 100.5, 120.5, 122.6, 123.7, 124. 4, 124.5, 138.7, 138.9, 145.8, 147.4, 164.7, 174.9 ppm; HRMS-ESI, m/z (Figure S13): Found: 333.0722 [M+H]^+^; Calcd for [C_16_H_17_N_2_O_2_S_2_]^+^ 333.0726; Found: 355.0544 [M+Na]^+^; calcd for [C_16_H_16_N_2_O_2_S_2_Na]^+^: 355.0545; Anal. Calcd. for C_16_H_16_N_2_O_2_S_2_ C, 57.81; H, 4.85; N, 8.43; Found C, 57.87; H, 4.80; N, 8.51.

*5-Carboxy-6-methyl-4-phenyl-3,4-dihydropyrimidin-2(1H)-thione***16b** [53]. By NaOH hydrolysis of the ethylester **1b** according to the literature [12]; 0.199 g, 80%, yellowish solid, m.p. 204–206 °C; IR (nujol): 3240, 1685, 1460, 1176, 689 cm^−1^; ^1^H-NMR (500 MHz, DMSO-d_6_): δ 2.28 (s, 3H, C(6)CH_3_), 5.15 (s, 1H, H-4), 7.20-7.35 (m, 5H, PhH), 9.57 (s, 1H, H-3), 10.22 (s, 1H, H-1) ppm; ^13^C-NMR (125.68 MHz, DMSO-d_6_): 17.9, 53.5, 98.6, 127.6, 128.4, 129.5, 144.8, 146.9, 173.7, 178.3; ESI-MS, m/z: 249.3 [M+H]^+^; Anal. Calcd. for C_12_H_12_N_2_O_2_S C, 58.05; H, 4.87; N, 11.28; Found C, 58.17; H, 4.80; N, 11.34.

#### 4.2.2. General Method for the Synthesis 3,4-Diihydropyrimidin-2(1*H*)-Imines **1c**–**3c**, **9c**, and **10c**

Compounds **1c**–**3c**, **9c**, and **10c** were prepared using a literature described protocol [29]. To a 0.5 M EtOH solution of the appropriate aldehyde (1mmol), β-ketoester (1.1 mmol), guanidine hydrochloride (1.5 mmol), and NaHCO_3_ (4 mmol) were added and the mixture irradiated at 120 °C for 10 min under microwaves conditions. After cooling to room temperature, the mixture was added to cold water to dissolve NaHCO_3_, and left at 5 °C for 30 min. The solid residue was filtered, washed with cold water, and finally triturated with diisopropyl ether or crystallized from hexane/ethyl acetate (3:1). Full characterization of these compounds is reported in [29].

#### 4.2.3. Synthesis of 5-Carboxy-4-Phenyl-6-Methyl-3,4-Dihydropyrimidine-2(1*H*)-Imine **16c**

Compound **16c** was prepared in 47% yield by the following procedure, involving steps (a) and (b).

(a) *Synthesis of 5-benzyloxycarbonyl-6-methyl-4-phenyl-3,4-dihydropyrimidin-2(1H)-imine***17c**. From benzaldehyde, benzylacetoacetate, guanidine hydrochloride under the same conditions as above [38]; 0.151 g, 47%; white solid, m.p. 168–170 °C; IR: 3359, 3500–2500 (broad), 1701, 1629, cm^−1^; ^1^H-NMR (500 MHz, CDCl_3_) (Figure S14): δ 2.20 (s, 3H, C(6)CH_3_), 4.92, 4.98 (AB system, *J* = 12.6 Hz, PhCH_2_), 5.22 (s, 1H, H-4), 6.21 (bs, 2H, H-1 and C=NH), 7.09-7.29 (m, 10H, 2xPhH) 7.33 (s, 1H, H-3) ppm; ^13^C-NMR (125.68 MHz, CDCl_3_) (Figure S15): δ 24.2, 53.0, 64.3, 96.9, 126.4, 127.4, 127.7, 127.8, 128.6, 137.6 146.8, 156.0, 162.4, 166.2 ppm; HRMS-ESI, m/z (Figure S16): Found: 322.1544 [M+H]^+^; Calcd for [C_19_H_20_N_3_O_2_]^+^ 322.1550.
(b) *Hydrogenolysis* of **17c**. A MeOH solution of the benzylester **17c** (0.100 g, 3.1 mmol) was added of 20 mg 10% Pd/C. The mixture was stirred overnight under a H_2_ atmosphere, then the solvent was removed in vacuo, to give the corresponding carboxylic acid **18c** (0.109 g) in a quantitative yield, m.p. 175–176 °C. IR: 3700–2300 (broad), 1700–1600 (multiple bands) cm^−1^; ^1^H-NMR (500 MHz, MeOD) (Figure S17): δ 2.22 (s, 3H, C(6)CH_3_), 5.50 (s, 1H, H-4), 6.16 (br, 2H, H-3 and C=NH), 7.15–7.30 (m, 5H, PhH), 7.39 (s, 1H, H-1) ppm; ^13^C-NMR (125.68 MHz, MeOD) (Figure S18): δ 21.5, 55.2, 109.0, 126.4, 127.8, 128.5, 145.6, 153.3, 160.0, 175.0 ppm; HRMS-ESI, m/z: Found: 232.1082 [M+H]^+^; Calcd for [C_12_H_14_N_3_O_2_]^+^ 232.1081; Anal. Calcd. for C_12_H_13_N_3_O_2_ C, 62.33; H, 5.67; N, 18.17; Found C, 62.37; H, 5.80; N, 18.08.

### 4.3. BACE1 Inhibition Assay

CE1 substrate (Arg-Glu(EDANS)-(Asn^670^,Leu^671^)-Amyloid β/A4 Protein Precursor_770 _(668-675)-Lys(DABCYL)-Arg trifluoroacetate salt) was purchased from Bachem. (Art. N. 4033536.000). Recombinant human β-Secretase, expressed in HEK 293 cells (C-terminal FLAG tagged), extracellular domain, ≥10,000 units/mg protein, was purchased from Aldrich.

A stock solution of the enzyme was prepared by diluting 5 µL of the commercial enzyme preparation in 995 µL of 20 mM acetate buffer at pH = 4.5, containing 4% DMSO. The solution was kept at 4 °C for 2 h and further 30 min at room temperature to allow folding of the protein, before starting the kinetic assay. In total, 200 μL of the enzyme solution were placed in a square fluorimetric cuvette (5 mm optical path) and 2 μL of a 10 mM DMSO solution of the substrate were added. The fluorescence emission was recorded for 30 min at 345 nm excitation and 505 nm emission (excitation and emission slits: 10 nm). The emission increases linearly during this time, and the slope obtained by plotting the emission vs. time was taken as the non-inhibited enzyme reaction rate. In total, 2 μL of a solution of the inhibitor were then added, and the reaction was followed again for 30 min, to measure the rate of the inhibited reaction. The inhibitor solutions were prepared from 10mM stock solutions of each inhibitor in DMSO, further diluted to 1 mM, 0.1 mM, 0.5 mM, 50 μM, 25 μM, and 5 μM mother solutions. Each mother solution was finally diluted 100 times when added to the cuvette. The measured inhibited rates were then plotted vs. the log of the inhibitor concentrations. The experimental points were fitted to a tetraparametric logistic function (Sigma Plot 13, SPSS inc) to obtain the IC_50_ values. The experiments were repeated three times for each inhibitor.

The assay was validated with a reference inhibitor (CAS 797035-11-1) purchased from Sigma Aldrich: theor. IC_50_ 15 nM [54]; found IC_50_ 19 nM.

### 4.4. Docking Studies

To build the models, file 2Q15 was downloaded from the Protein Data Bank, and after adding all the hydrogens and adjusting the protonation state of ionizable residues to pH 5, the structure was relaxed by an energy minimization of 50,000 steps of steepest descent method, keeping initially frozen the protein backbone and then allowing the whole structure to relax. The minimization was carried out with the OPLS3 forcefield [55] as implemented in the Schrödinger suite [56]. The ligands were then manually docked to the enzyme’s binding site, and a starting geometry was obtained by superimposing the guanidine group of each inhibitor onto that of the Baxter inhibitor in the relaxed structure of the model. At least two starting models for each inhibitor were obtained by different superimposition modes. The starting models of each inhibitor-BACE-1 complex were then thermalized by a molecular dynamic run carried out in the NTP ensemble at 300 °K for 100 ns. The dynamics outcomes were then analyzed, and the lowest energy conformation for each run was chosen for the final optimization of the complexes, carried out as described above for the initial model, using the conjugate gradient optimization algorithm. Binding energies were calculated according to Equation (2).

(2) $$\Delta E_{b}={\;E}_{\mathrm{EI}}-E_{E}^{0}-E_{I}^{0}\;$$

where E_EI_ is the OPLS3 energy of the optimized enzyme–inhibitor complex, $E_{E}^{0}$ is the OPLS3 energy of the empty enzyme after relaxation to its closest energy minimum (the same for each model), and $E_{I}^{0}$ is the OPLS3 energy of the free ligand in its absolute minimum energy conformation as obtained from a conformational search. In this preliminary study all the optimizations were carried out in vacuo and no solvation effects were considered.

### 4.5. Prediction of ADME Properties

Chemicalize was used for prediction of LogP and LogD, August 2020, developed by ChemAxon [57]. Predictions of Blood brain barrier permeation (BBB +/−) based on the BOILED-Egg model [42] were obtained with the SwissADME web tool [58].

## Figures and Tables

**Table 1:** Synthesis^1^ and BACE-1 inhibitory activity of DHPMs and their thia and imino derivatives obtained as in Scheme 1.

Dihydropyrimidine			Yield (%)	IC_50_ (μM)	LogP^2^	Dihydropyrimidine			Yield (%)	IC_50_ (μM)	LogP^2^
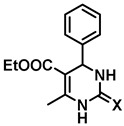						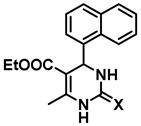					
	**1a**	X=O	82	2.8 ± 0.1	1.2		**9a**	X=O	89	3.1 ± 0.4	2.2
	**1b**	X=S	85	1.5 ± 0.4	2.1		**9b**	X=S	87	0.3 ± 0.1	3.1
	**1c**	X=NH	83	0.5 ± 0.1	1.5		**9c**	X=NH	88	1.5 ± 0.4	2.5
					(−0.8)^3^						(0.1)^3^
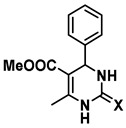						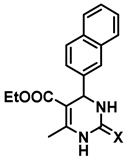					
	**2a**	X=O	83	0.32 ± 0.05	0.9		**10a**	X=O	87	0.50 ± 0.05	2.2
	**2b**	X=S	87	0.85 ± 0.03	1.8		**10b**	X=S	88	1.1 ± 0.1	3.1
	**2c**	X=NH	80	0.52 ± 0.11	1.1		**10c**	X=NH	90	0.2 ± 0.1	2.5
					(−1.2)^3^						(0.2)^3^
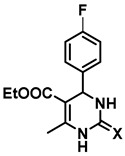						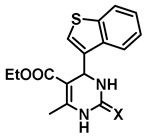	**11a**	X=O	73	71.3 ± 1.2	2.1
	**3a**	X=O	70	1.7 ± 0.4	1.4						
	**3b**	X=S	68	0.24 ± 0.05	2.3						
	**3c**	X=NH	70	0.30 ± 0.15	1.6						
					(−0.6)^3^						
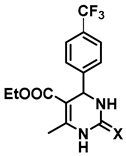	**4a**	X=O	81	0.6 ± 0.3	2.1	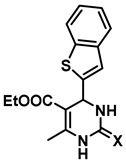	**12a**	X=O	85	1.35 ± 0.10	2.3
							**12b**	X=S	73	0.7 ± 0.1	3.1
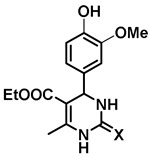	**5a**	X=O	78	0.65 ± 0.03	0.8	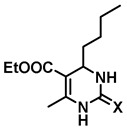	**13a**	X=O	50	2.3 ± 0.2	1.3
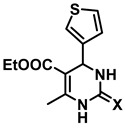	**6a**	X=O	58	3.2 ± 0.3	1.0	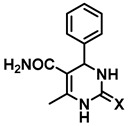	**14a**	X=O	47	1.6 ± 0.1	−0.3
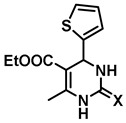	**7a**	X=O	61	2.2 ± 0.2	1.2	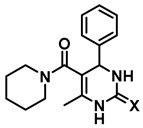	**15a**	X=O	60	1.6 ± 0.1	−0.1
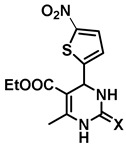	**8a**	X=O	50	1.2 ± 0.4	1.3	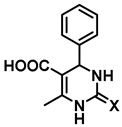	**16a**	X=O	45^4^	34.0 ± 8.0	−2.9^3^
							**16b**	X=S	80^4^	1.2 ± 0.3	−1.9^3^
							**16c**	X=NH	47^5^	0.7 ± 0.2	−0.91

^1^ Conditions: X=O,S, solvent-free, NH_4_Cl, 100 °C, 3–12 h; X = NH: EtOH, NaHCO_3_ (4 eqv), MW 120°, 10 min [29]; ^2^ LogP and LogD (for ionizable compounds) values were calculated with Chemicalize by ChemAxon. ^3^ LogD at pH 7.4. ^4^ By NaOH hydrolysis of esters **1a** and **1b**. ^5^ By H_2_/Pd-C hydrogenolysis of the corresponding benzyl ester.

**Table 2:** Calculated relative binding energies and experimental IC_50**.**_

Inhibitor	Experimental IC_50_^1^ (nM)	Rel ΔE_b_^2^ (Kcal mol^−1^)
**(*R*)-10c**	200	0
**(*S*)-10c**		5.2
**(*R*)-9c**	150	5.6
**(*R*)-1c**	500	2.3

^1^ racemic mixture. ^2^ relative to the binding energy of **(*R*)-10c**.

## Supplementary Materials

The following are available online. Figures S2–S19: ^1^H-NMR, ^13^C-NMR and MS spectra of literature unknown compounds; Figures S20 and S21 supplementary docking figures.
