# Supplementary material for: One Pot Synthesis of Micromolar BACE-1 Inhibitors Based on the Dihydropyrimidinone Scaffold and Their Thia and Imino Analogues
Source: Molecules. 2020 Sep 10;25(18):4152. doi: 10.3390/molecules25184152 (PMC7571164; doi:10.3390/molecules25184152)
Supplement: Supplementary file 1 [file molecules-25-04152-s001.pdf]

Supporting Information

One pot synthesis of µM BACE-1 inhibitors based on the dihidropyrimidinone scaffold and their thia and imino analogues

Jessica Bais[1], Fabio Benedetti[1], Federico Berti[1], Iole Cerminara[2], Sara Drioli[1], Fulvia Felluga[1*], Maria Funicello[2], Giorgia Regini[1], Mattia Vidali[1].

[1] Dipartimento di Scienze Chimiche e Farmaceutiche, Università di Trieste, via Licio Giorgieri, 1 34127 Trieste (Italy)

[2] Dipartimento di Scienze, Università della Basilicata, Viale dell'Ateneo Lucano, 10 85100 Potenza (Italy)

Content

^{1}H-NMR, ^{13}C-NMR and HRMS spectra of 9a, 12a,13a, 13b, 16c, 17c **S2 – S19**

Supplementary molecular docking images **S20,S21**

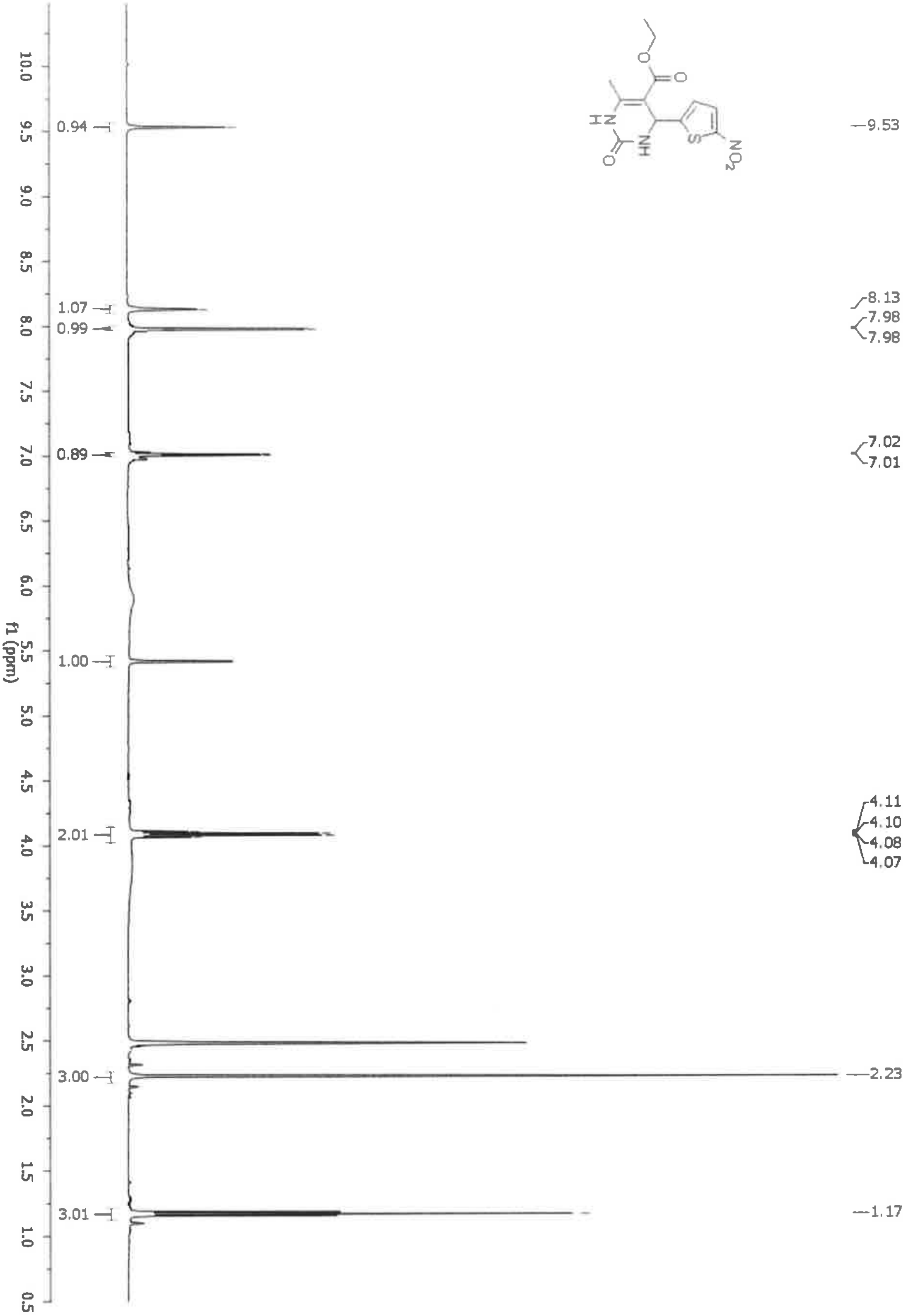
f1 (ppm)
0.94
1.07
0.99
0.89
1.00
2.01
3.00
3.01
9.53
8.13
7.98
7.98
7.02
7.01
4.11
4.10
4.08
4.07
2.23
1.17
NO2
NH
S
O

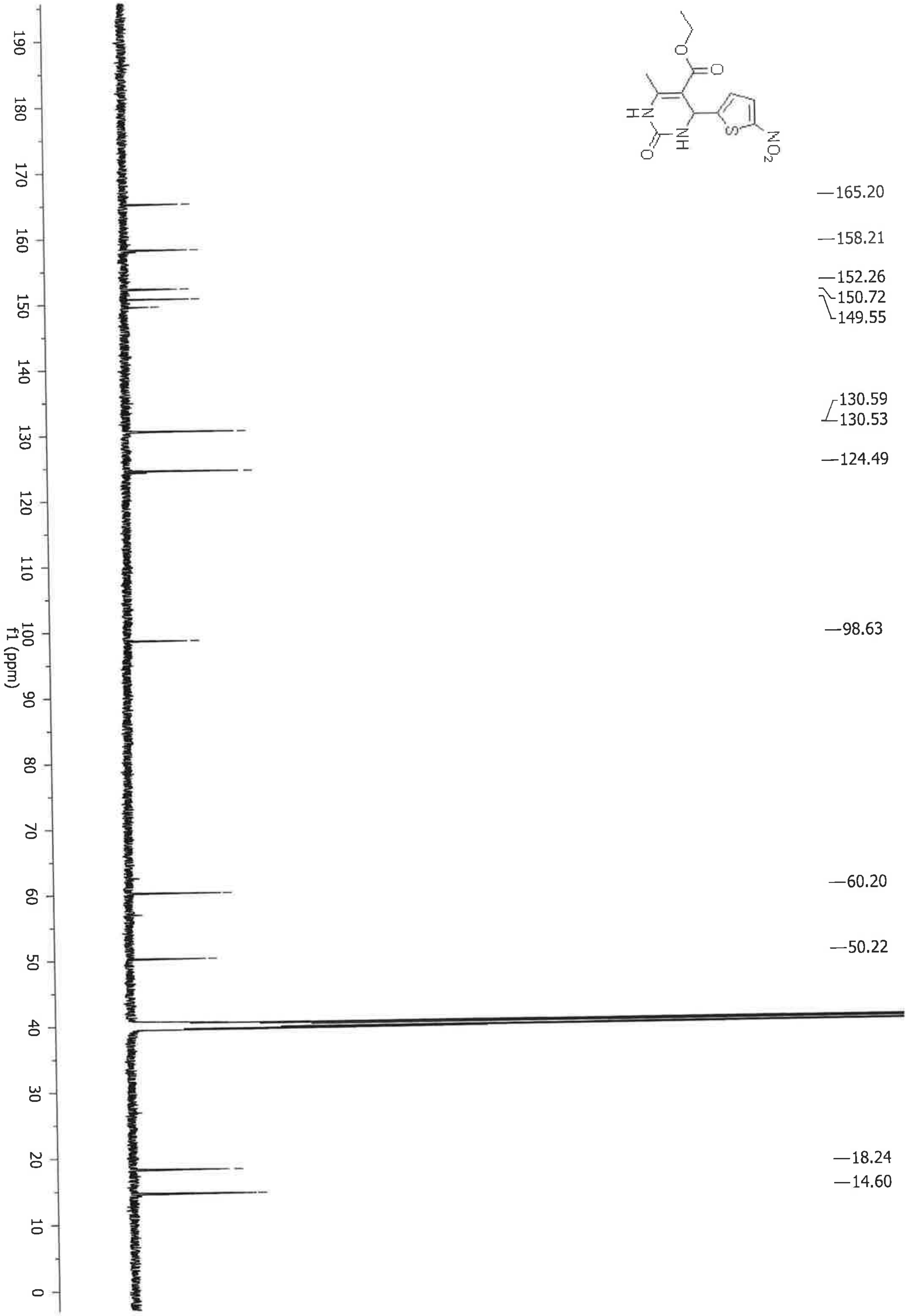

165.20
158.21
152.26
150.72
149.55
130.59
130.53
124.49
98.63
60.20
50.22
18.24
14.60
f1 (ppm)
NH
NO2
S

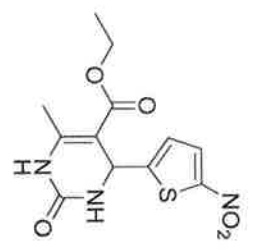
NO2
S
NH
N
H
O
O
O

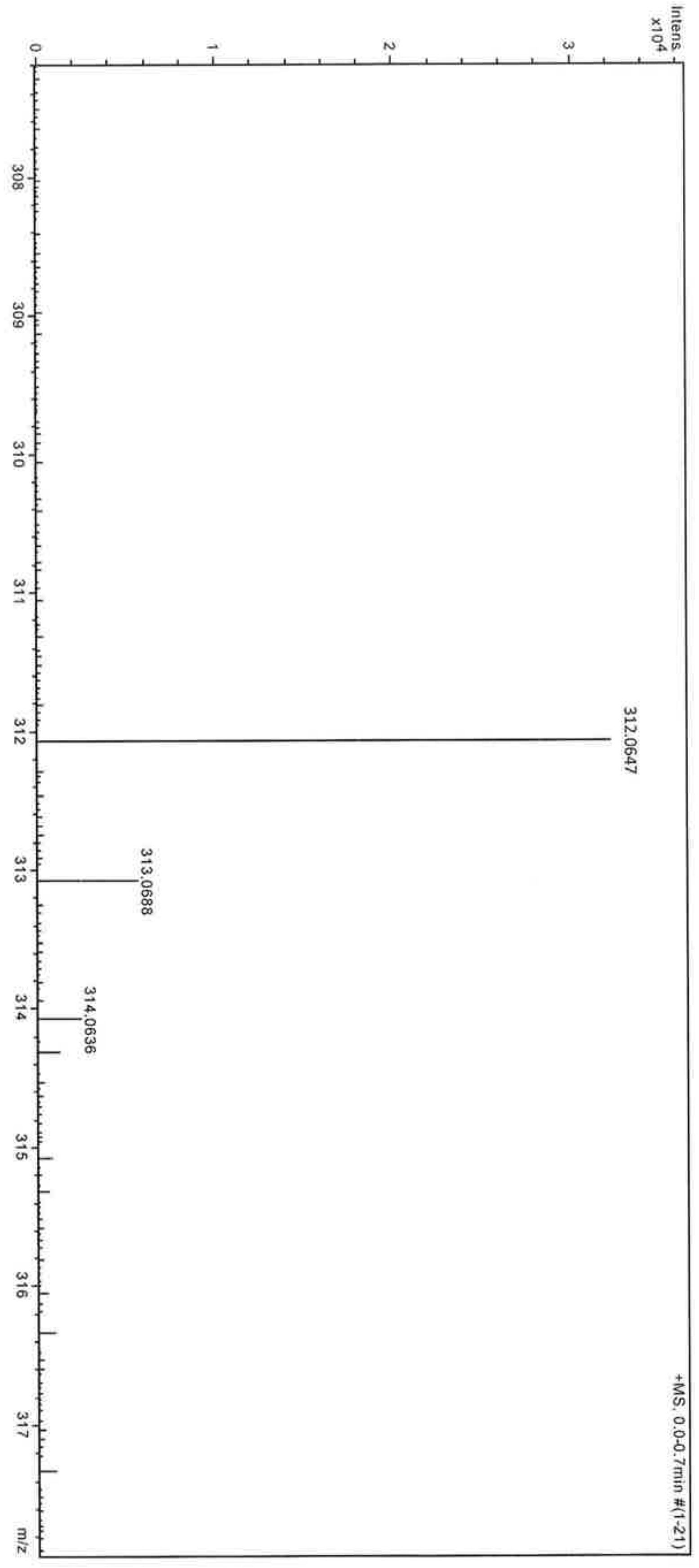
Intens.
x10^4
+MS, 0.0-0.7min #(1-21)
312.0647
313.0688
314.0636
m/z

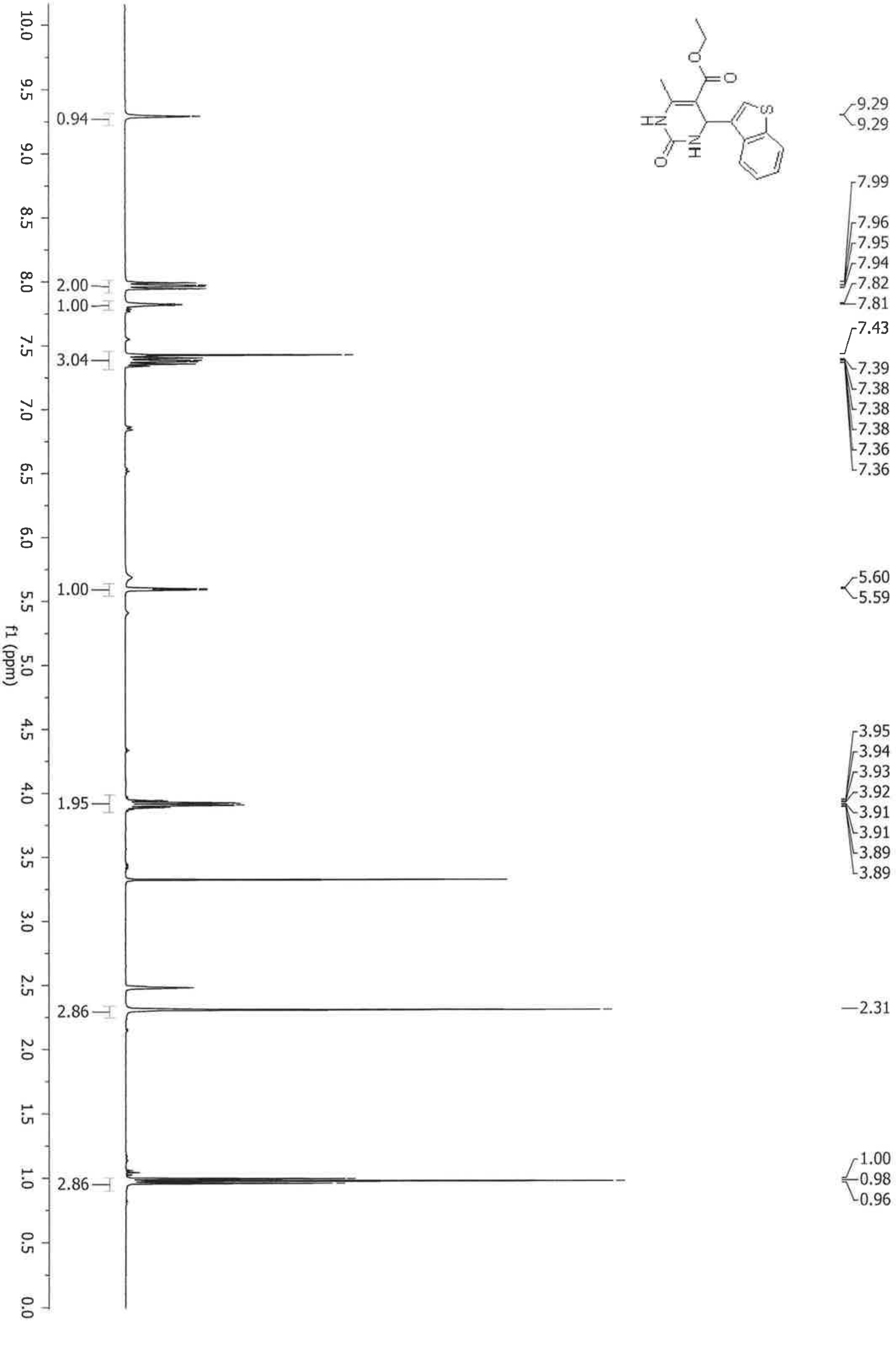
f1 (ppm)
0.94
2.00
1.00
3.04
1.00
1.95
2.86
2.86
9.29
9.29
7.99
7.96
7.95
7.94
7.82
7.81
7.43
7.39
7.38
7.38
7.38
7.36
7.36
5.60
5.59
3.95
3.94
3.93
3.92
3.91
3.91
3.89
3.89
2.31
1.00
0.98
0.96

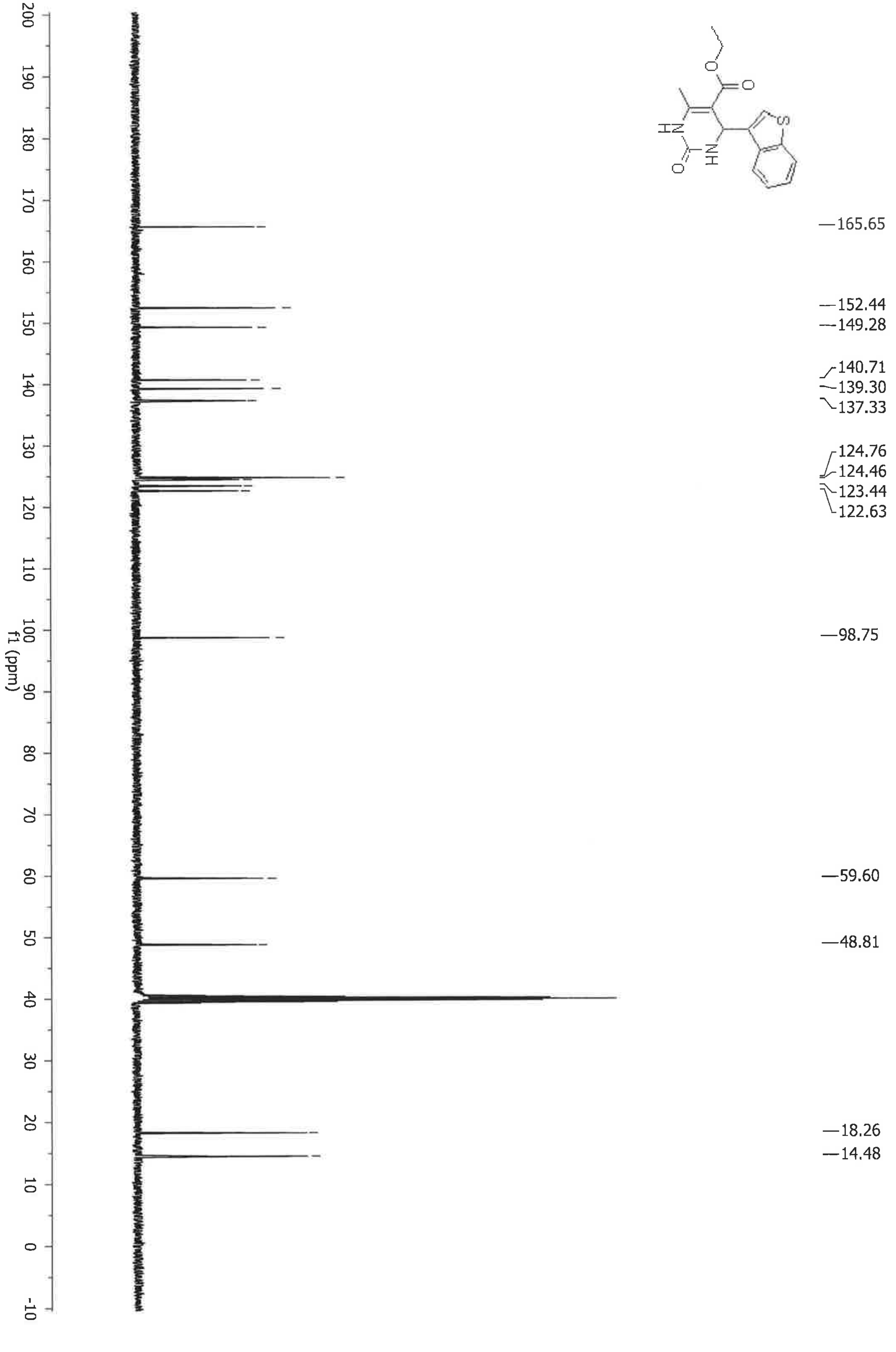
200
190
180
170
160
150
140
130
120
110
100
90
80
70
60
50
40
30
20
10
0
-10
f1 (ppm)
O
NH
H
N
O
O
S
165.65
152.44
149.28
140.71
139.30
137.33
124.76
124.46
123.44
122.63
98.75
59.60
48.81
18.26
14.48

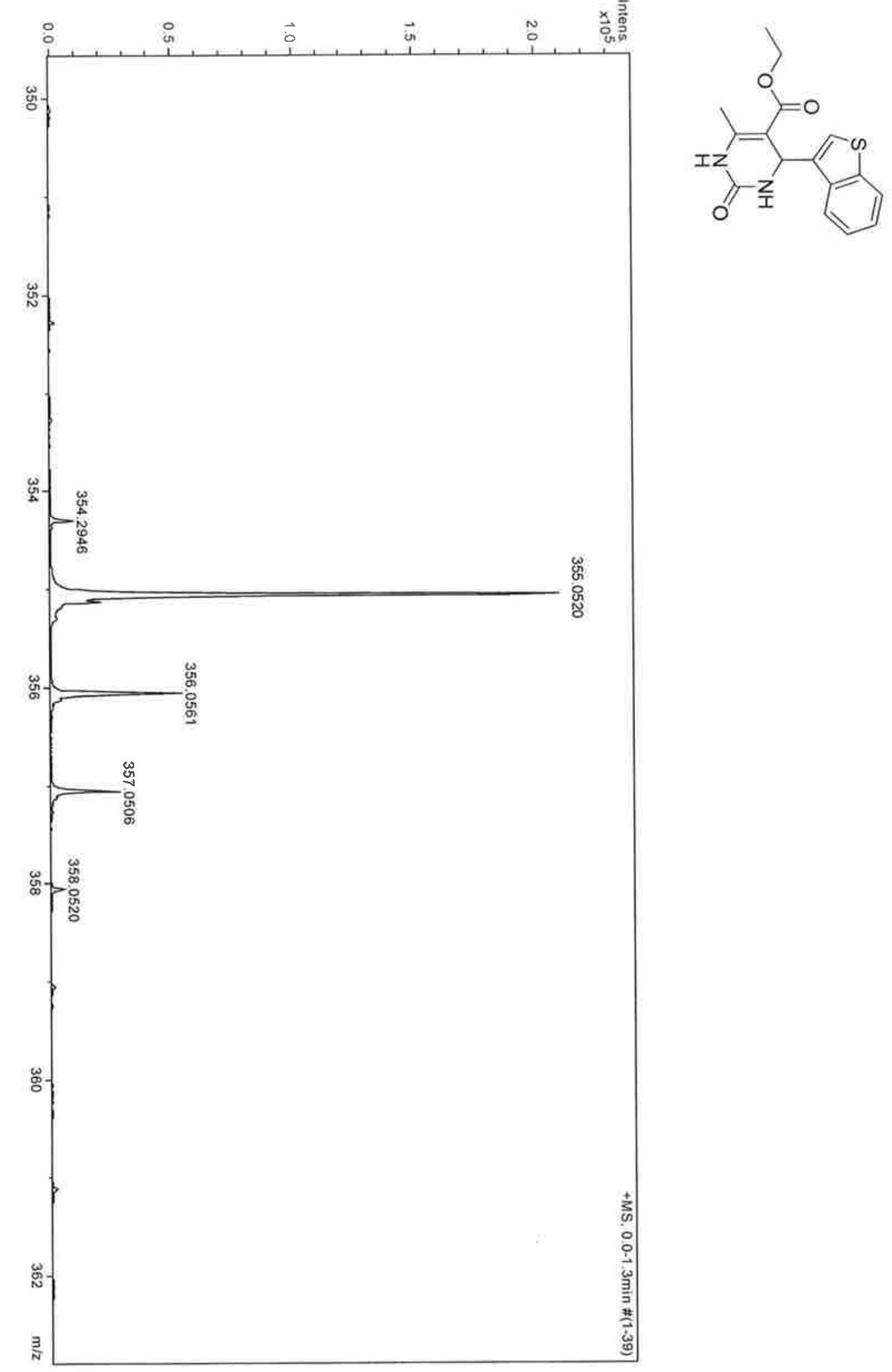
Intens.
x10^5
2.0
1.5
1.0
0.5
0.0
+MS, 0.0-1.3min #(1-39)
355.0520
356.0561
357.0506
354.2946
358.0520
350
352
354
356
358
360
362
m/z
S
NH
N
H
O
O
O

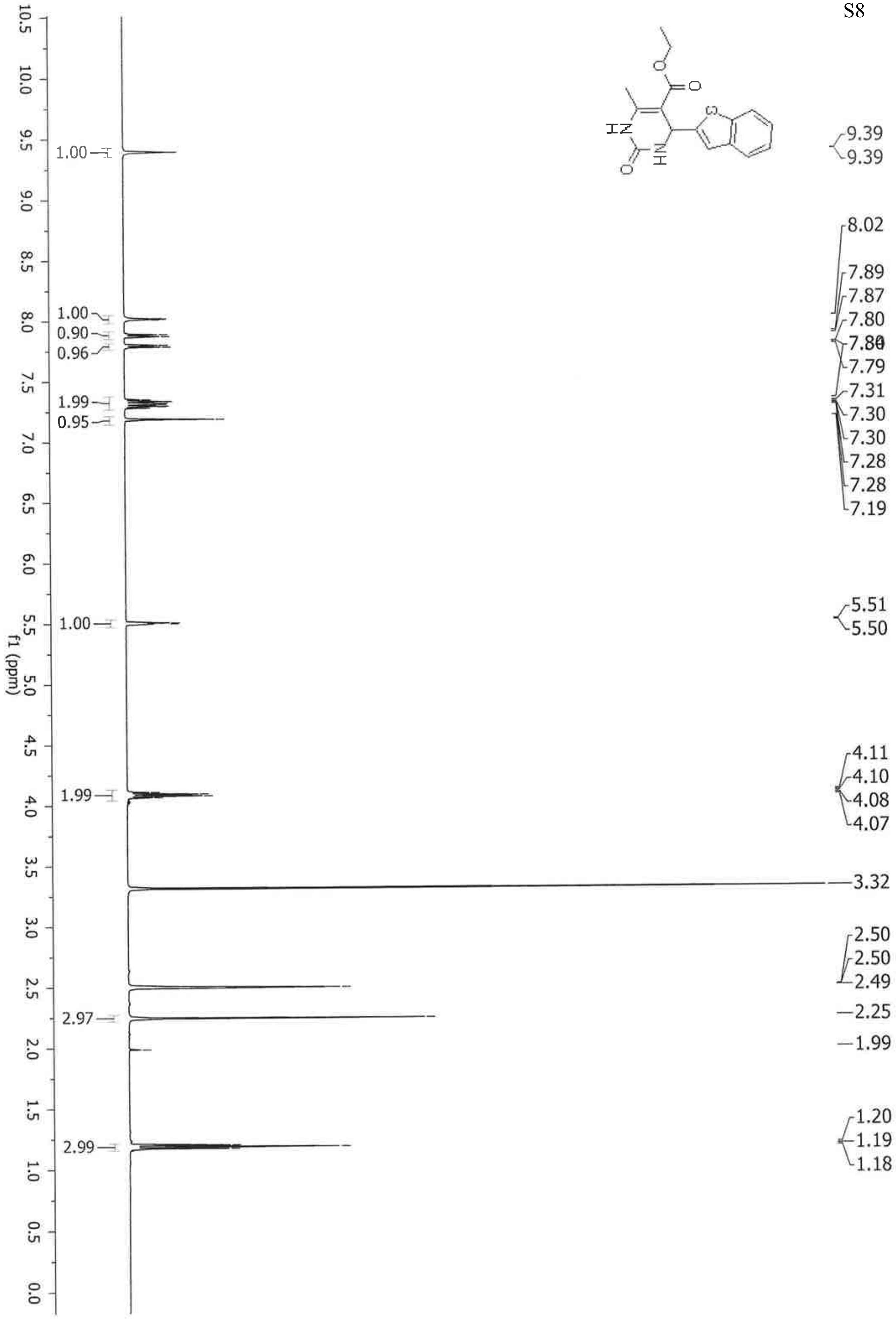

f1 (ppm)
9.39
9.39
8.02
7.89
7.87
7.80
7.80
7.79
7.31
7.30
7.30
7.28
7.28
7.19
5.51
5.50
4.11
4.10
4.08
4.07
3.32
2.50
2.50
2.49
2.25
1.99
1.20
1.19
1.18
1.00
1.00
0.90
0.96
1.99
0.95
1.00
1.99
2.97
2.99

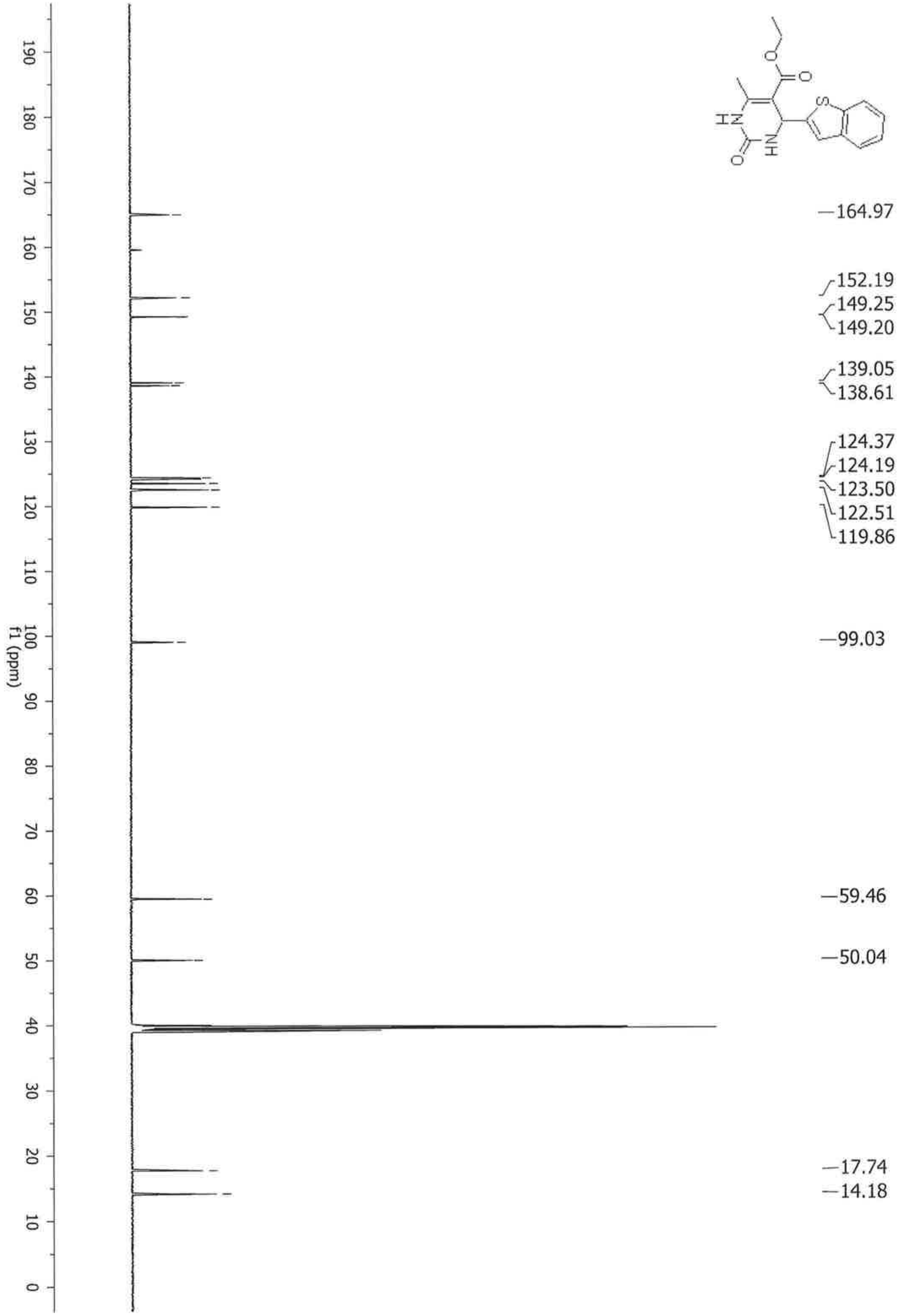
164.97
152.19
149.25
149.20
139.05
138.61
124.37
124.19
123.50
122.51
119.86
99.03
59.46
50.04
17.74
14.18
f1 (ppm)
H
N
NH
O
O
O
S

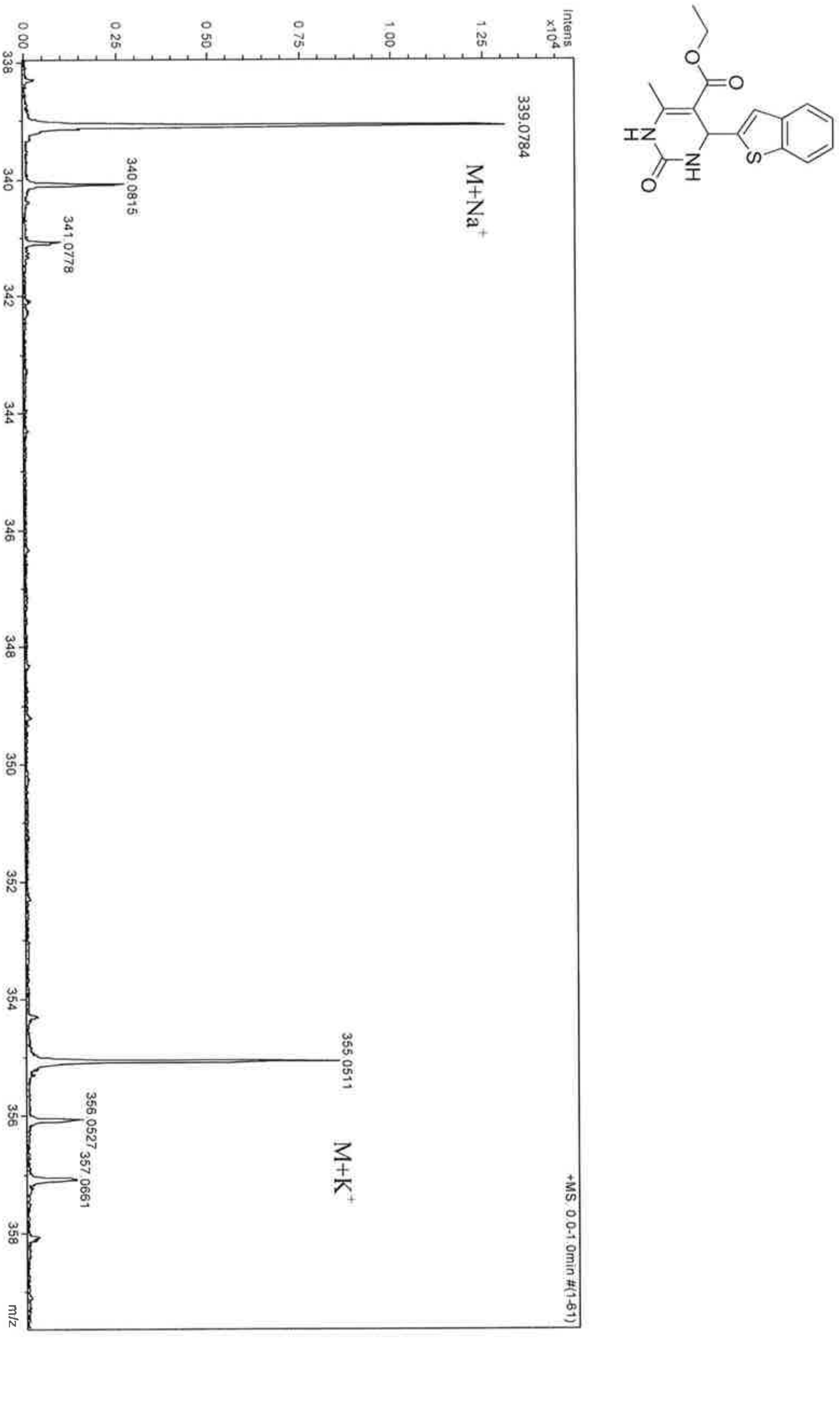

Intens
x10^4
+MS, 0.0-1.0min #(1-61)
339.0784
M+Na+
340.0815
341.0778
355.0511
356.0527
357.0661
M+K+
m/z

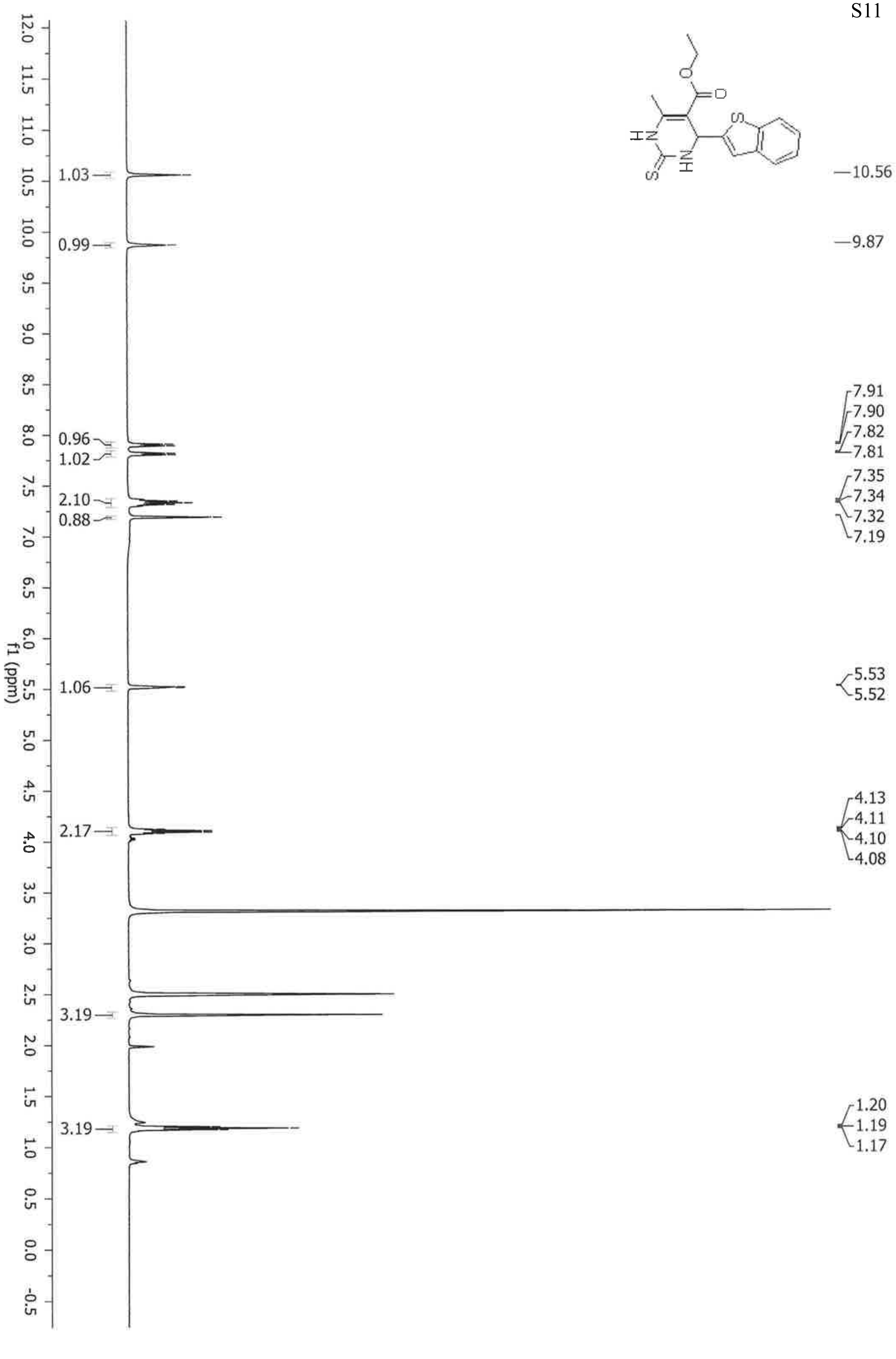

f1 (ppm)
1.03
0.99
0.96
1.02
2.10
0.88
1.06
2.17
3.19
3.19
10.56
9.87
7.91
7.90
7.82
7.81
7.35
7.34
7.32
7.19
5.53
5.52
4.13
4.11
4.10
4.08
1.20
1.19
1.17

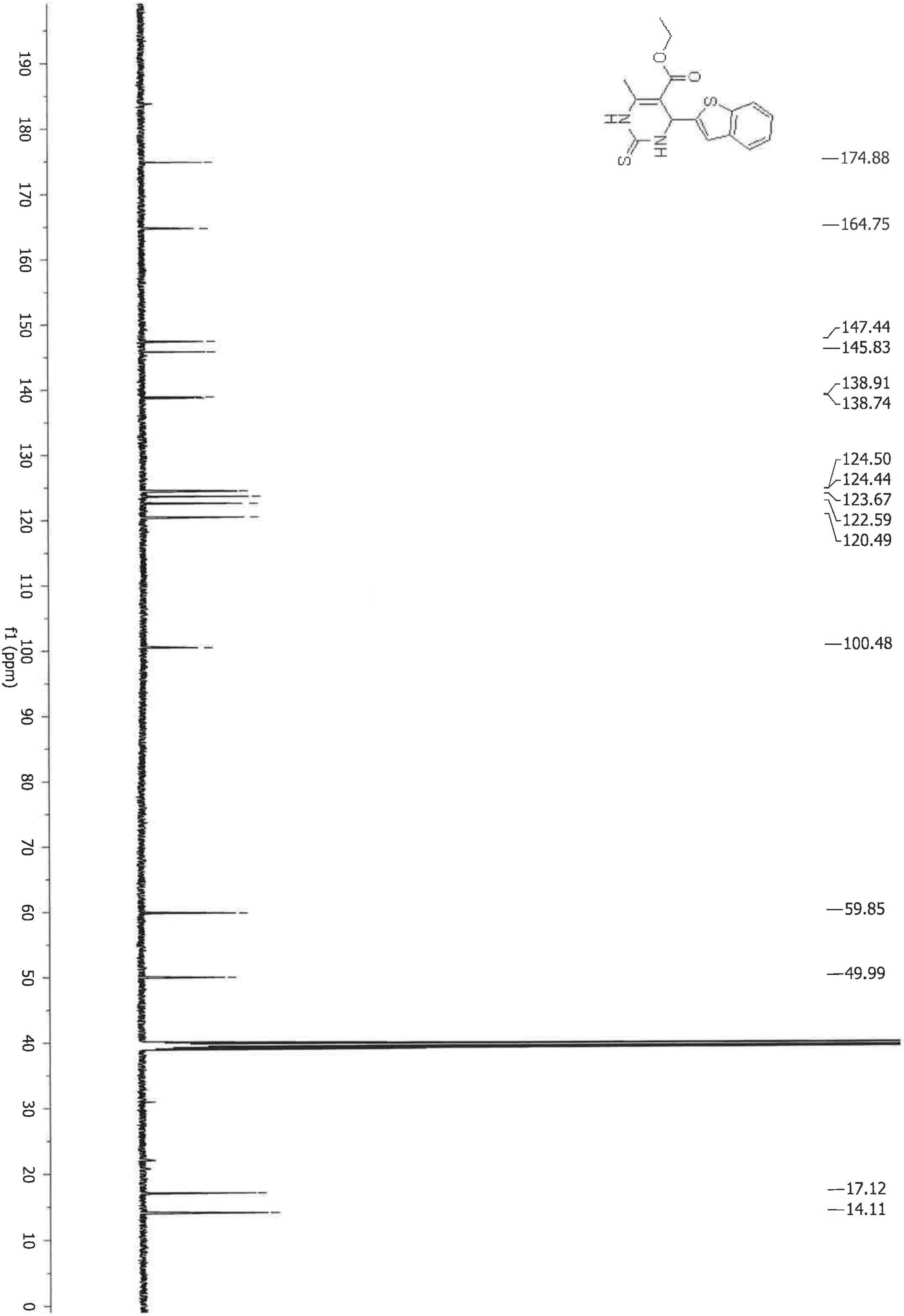
174.88
164.75
147.44
145.83
138.91
138.74
124.50
124.44
123.67
122.59
120.49
100.48
59.85
49.99
17.12
14.11
f1 (ppm)

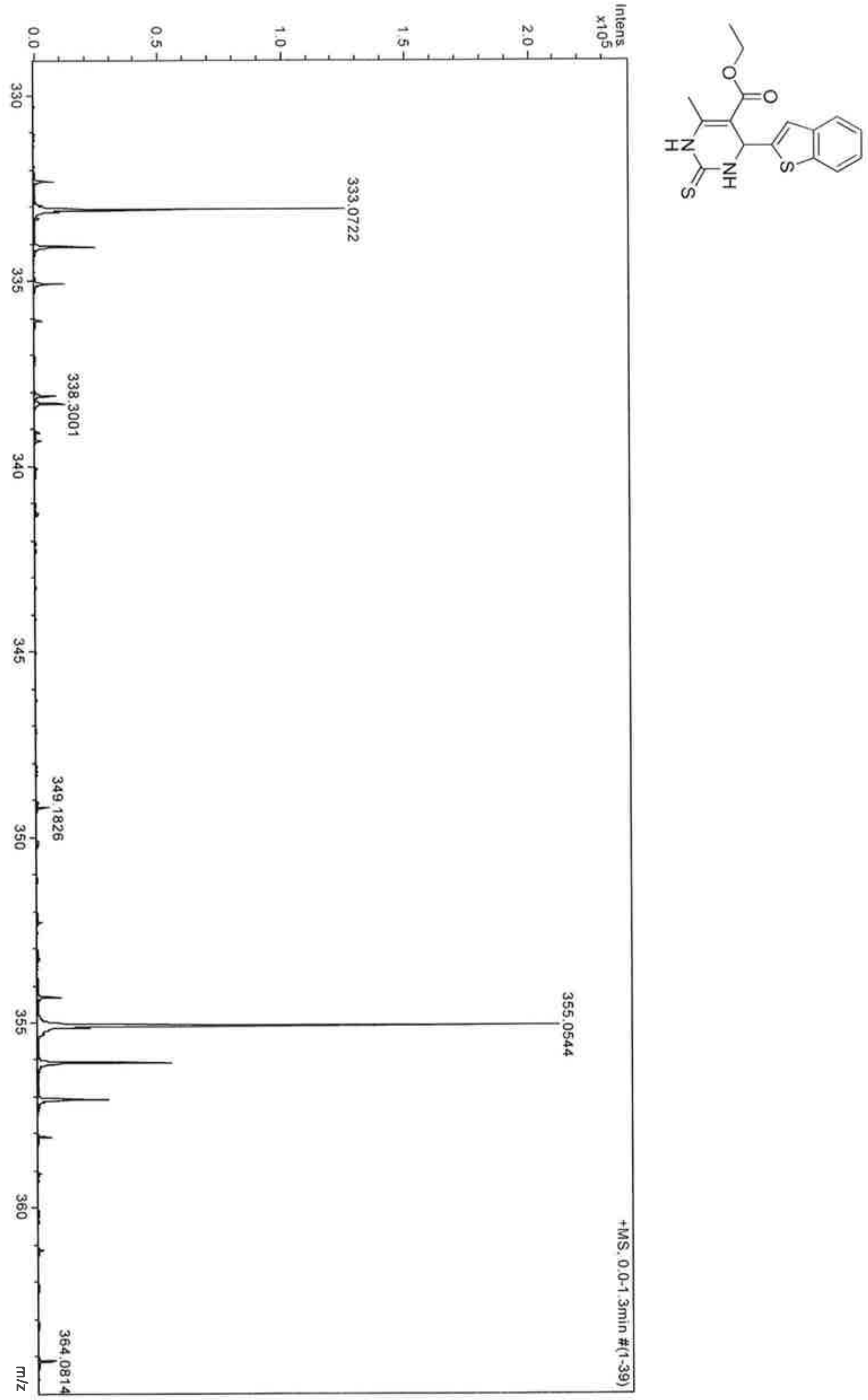
Intens.
x10^5
+MS, 0.0-1.3min #(1-39)
333.0722
338.3001
349.1826
355.0544
364.0814
m/z

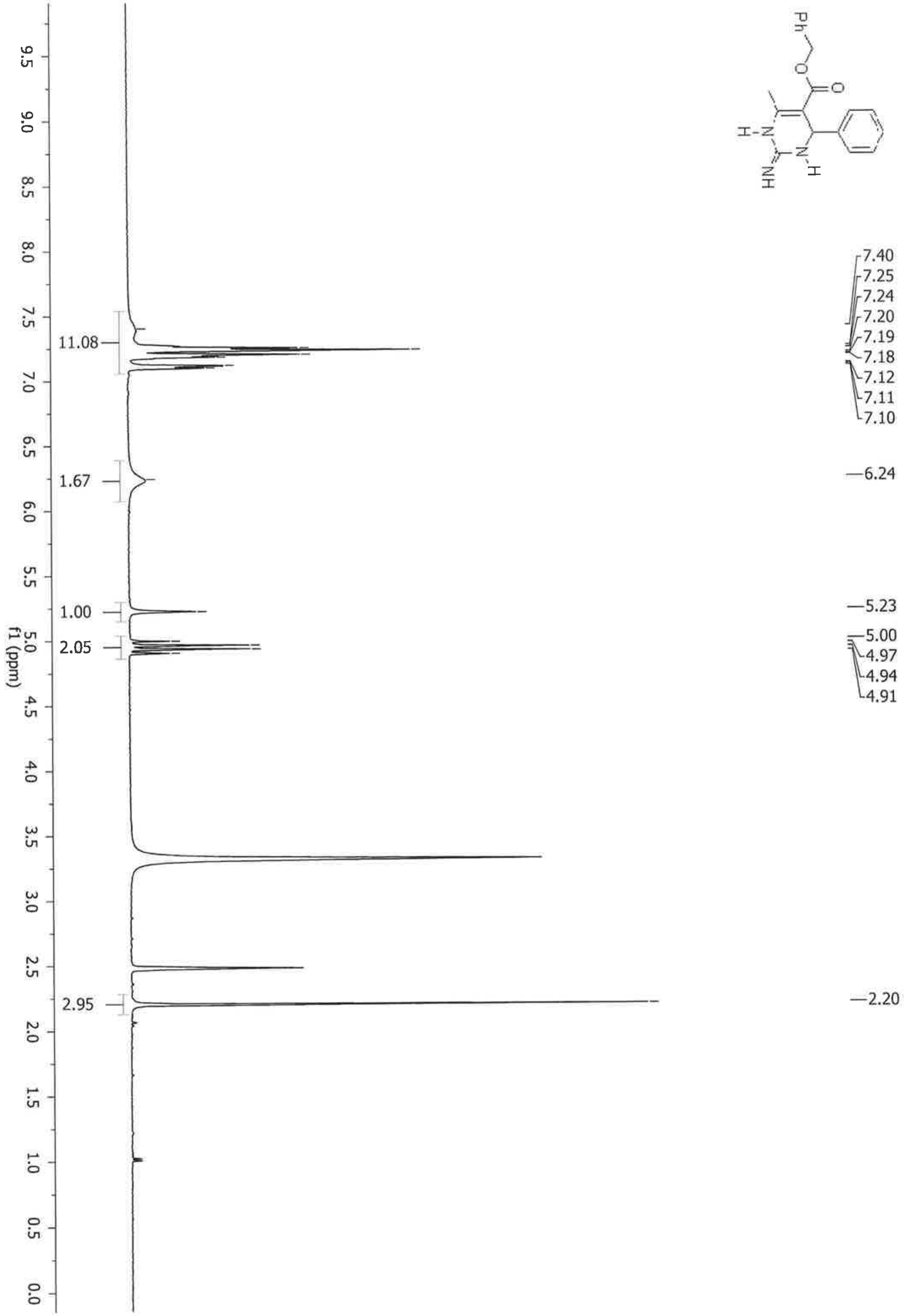

Ph
O
O
NH
N
H
N
H
7.40
7.25
7.24
7.20
7.19
7.18
7.12
7.11
7.10
6.24
5.23
5.00
4.97
4.94
4.91
2.20
11.08
1.67
1.00
2.05
2.95
9.5
9.0
8.5
8.0
7.5
7.0
6.5
6.0
5.5
5.0
4.5
4.0
3.5
3.0
2.5
2.0
1.5
1.0
0.5
0.0
f1 (ppm)

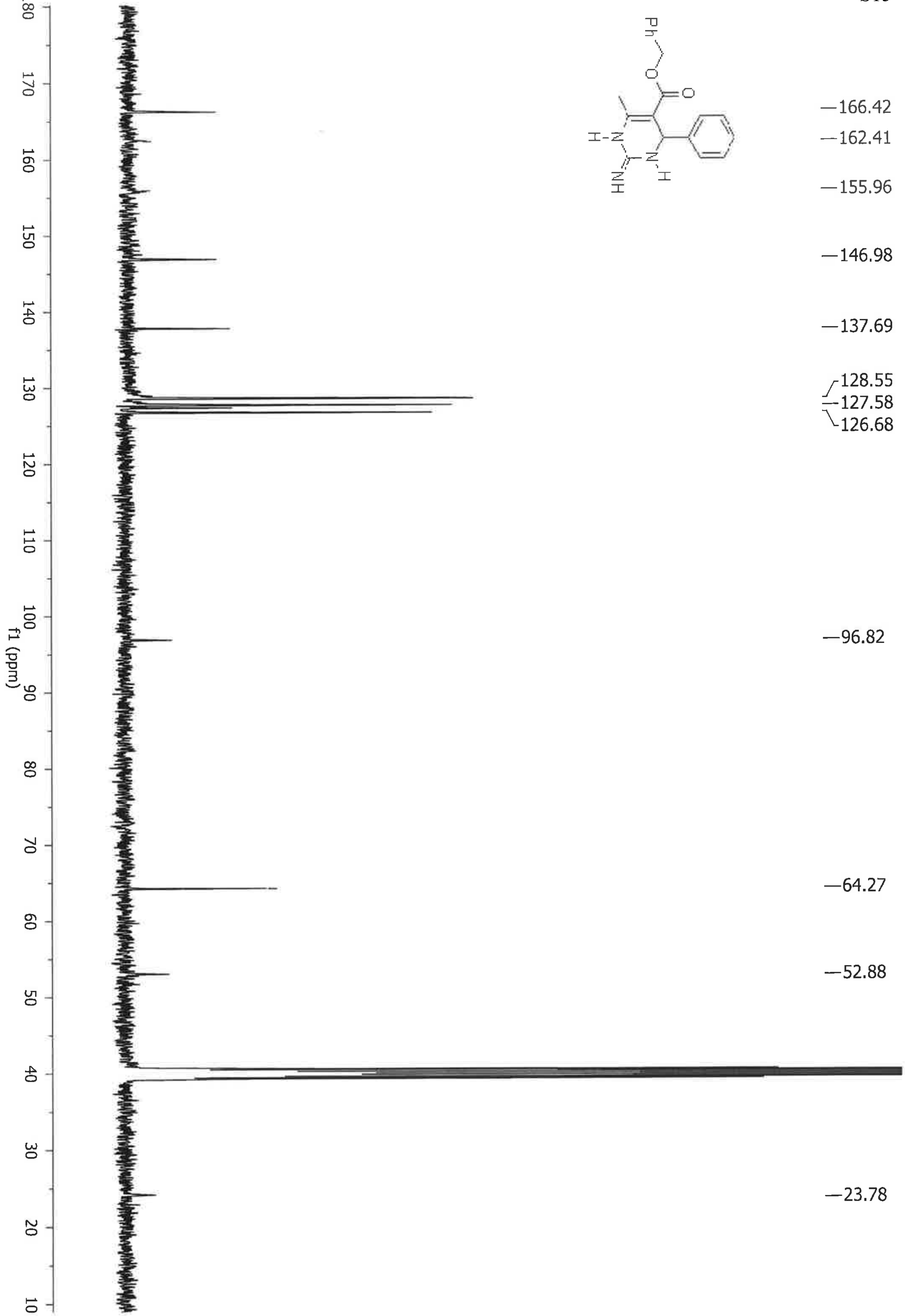
—166.42
—162.41
—155.96
—146.98
—137.69
128.55
127.58
126.68
—96.82
—64.27
—52.88
—23.78
f1 (ppm)
Ph
O
NH
N
H

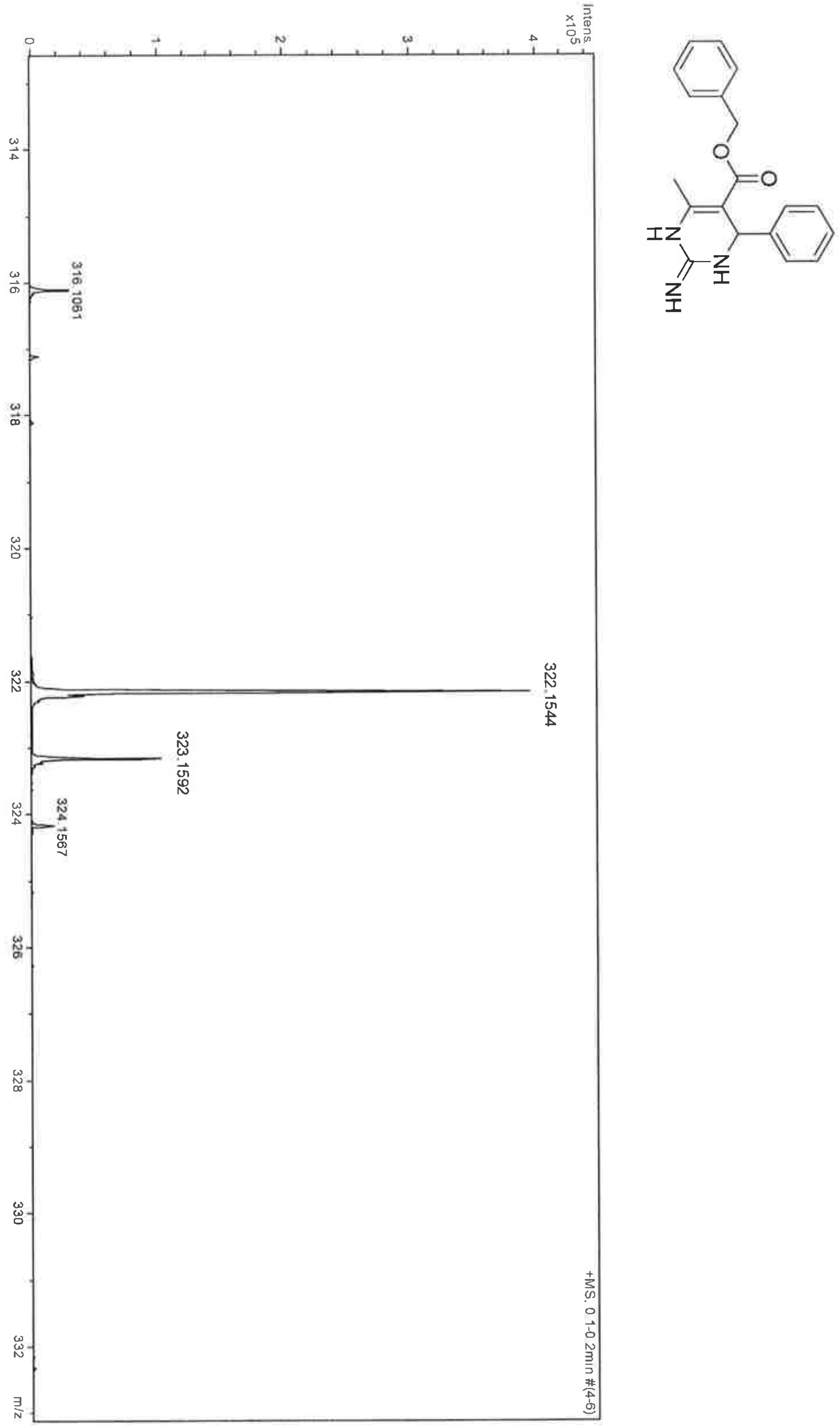

Intens.
x10^5
+MS, 0.1-0.2min #(4-6)
316.1061
322.1544
323.1592
324.1567
m/z
NH
N
H

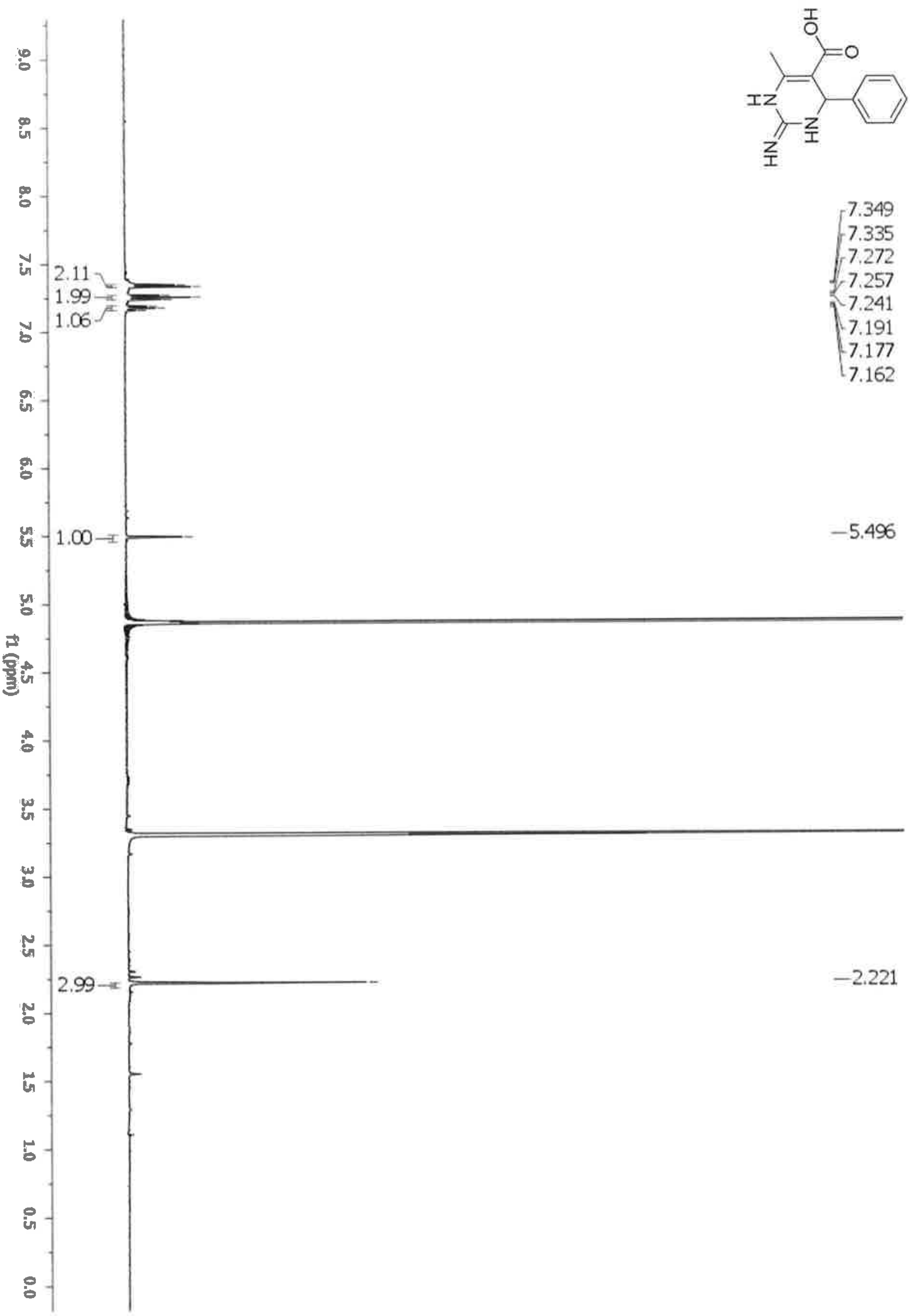

HO
O
HN
NH
H
N
7.349
7.335
7.272
7.257
7.241
7.191
7.177
7.162
5.496
2.221
2.11
1.99
1.06
1.00
2.99
9.0
8.5
8.0
7.5
7.0
6.5
6.0
5.5
5.0
4.5
4.0
3.5
3.0
2.5
2.0
1.5
1.0
0.5
0.0
f1 (ppm)

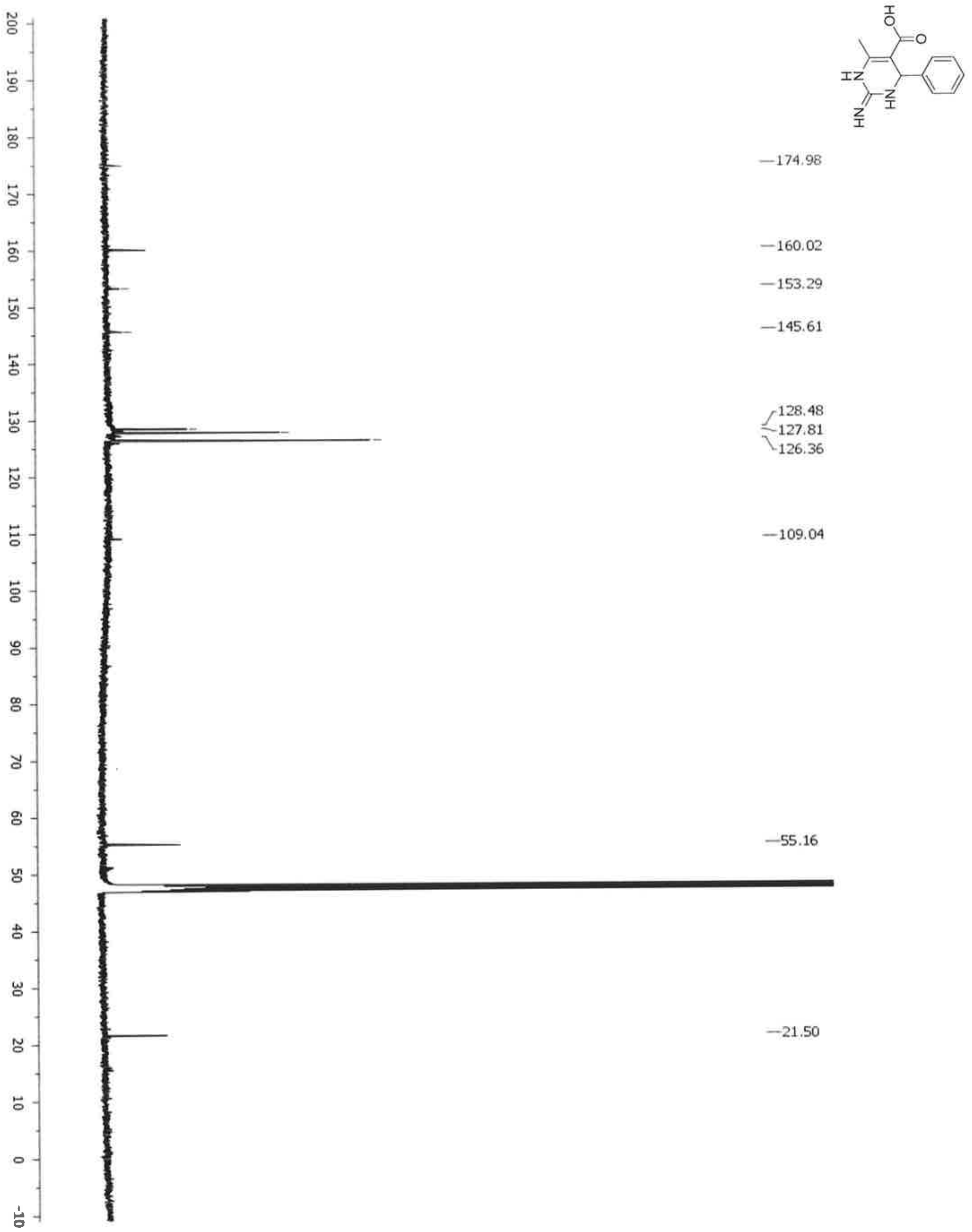
174.98
160.02
153.29
145.61
128.48
127.81
126.36
109.04
55.16
21.50

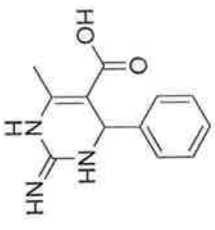

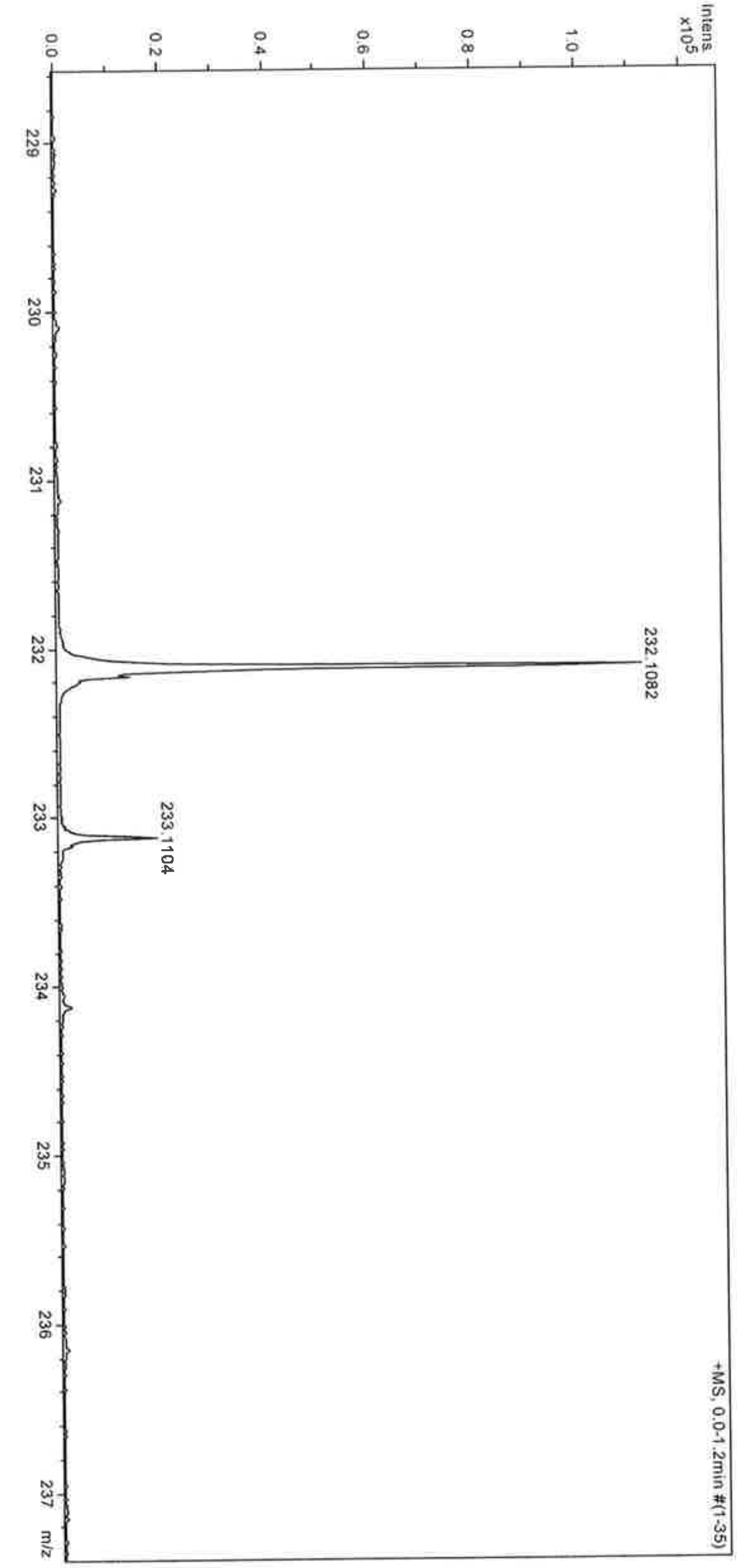
Intens.
x10^5
232.1082
233.1104
+MS, 0.0-1.2min #(1-35)
m/z

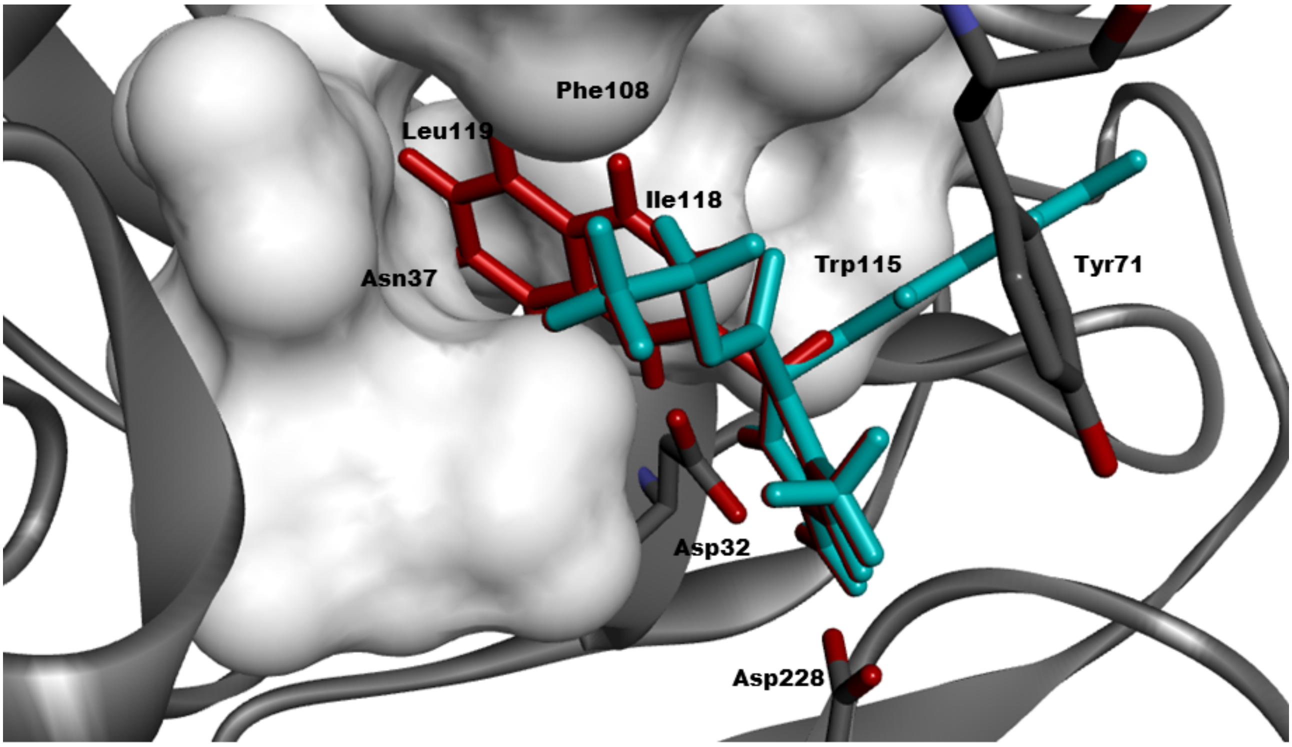

Supplementary figure 1: overlay of the *R* (blue) and *S* (red) enantiomers of inhibitor **10c** inside BACE-1 catalytic site. The structure has been optimized for the *R* enantiomer, and shows the bumping at the naphthalene ring of the *S* enantiomer when the guanidium group is constrained in the optimal position for hydrogen bonding to the aspartate diad.

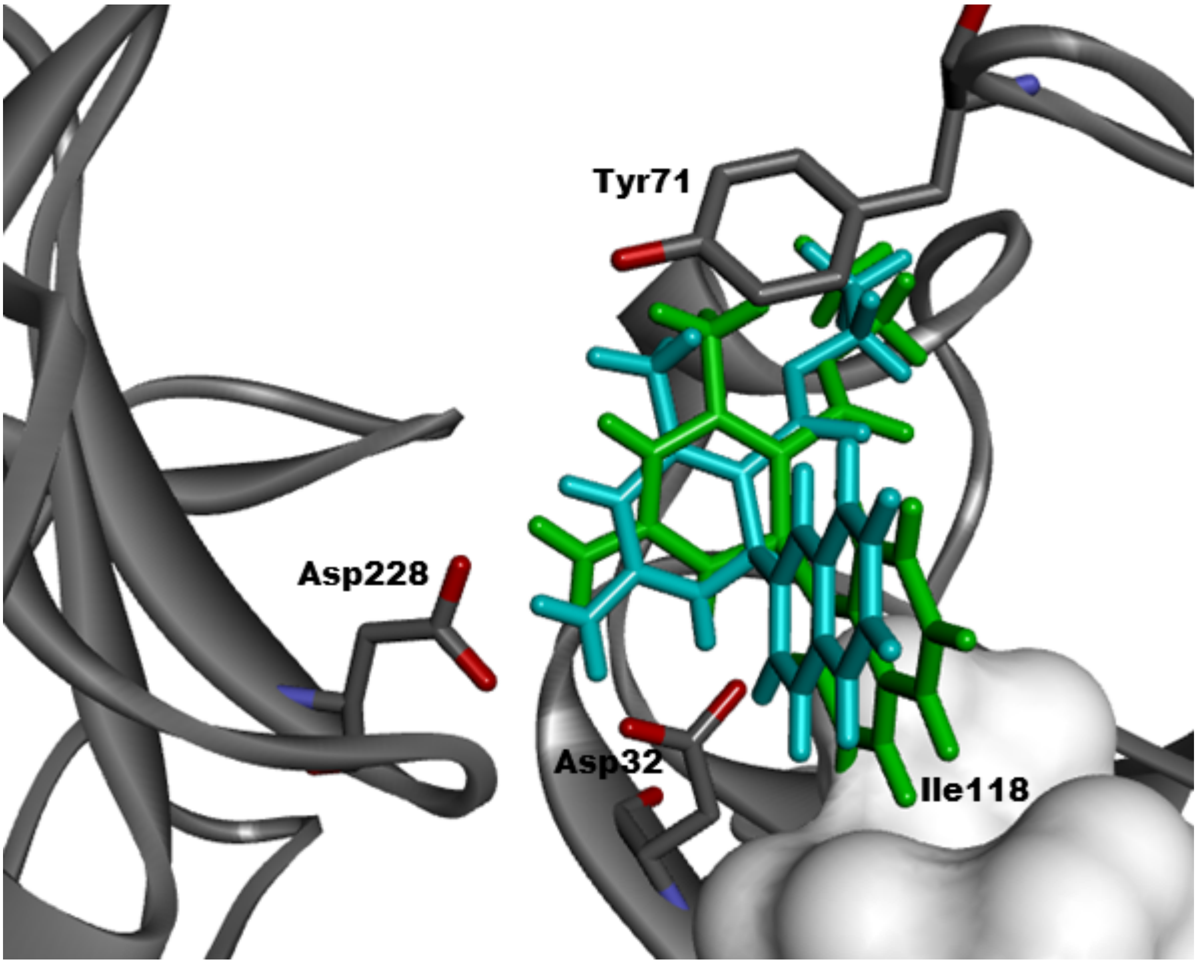

Supplementary figure 2: overlay of the optimized complexes of Bace-1 with inhibitors **(*R*)-10c** (blue) and **(*R*)-9c** (green)
